# Chitosan/hesperidin nanoparticles formulation: a promising approach against ethanol-induced gastric ulcers via Sirt1/FOXO1/PGC-1α/HO-1 pathway

**DOI:** 10.3389/fphar.2024.1433793

**Published:** 2024-09-09

**Authors:** Jawaher Abdullah Alamoudi, Thanaa A. El-Masry, Maysa M. F. El-Nagar, Enas I. El Zahaby, Kadreya E. Elmorshedy, Mohamed M. S. Gaballa, Samar Zuhair Alshawwa, Maha Alsunbul, Sitah Alharthi, Hanaa A. Ibrahim

**Affiliations:** ^1^ Department of Pharmaceutical Sciences, College of Pharmacy, Princess Nourah bint Abdulrahman University, Riyadh, Saudi Arabia; ^2^ Department of Pharmacology and Toxicology, Faculty of Pharmacy, Tanta University, Tanta, Egypt; ^3^ Department of Pharmaceutics, Faculty of Pharmacy, Delta University for Science and Technology, Gamasa, Egypt; ^4^ Department of Anatomy, Faculty of Medicine, Tanta University, Tanta, Egypt; ^5^ Department of Anatomy, King Khaled College of Medicine, Riyadh, Saudi Arabia; ^6^ Department of Pathology, Faculty of Veterinary Medicine, Benha University, Toukh, Egypt; ^7^ Department of Pharmaceutics, College of Pharmacy, Shaqra University, Shaqra, Saudi Arabia

**Keywords:** hesperidin, gastric ulcer, nano hesperidin, ethanol, oxidative stress, inflammation, Sirt-1, PGC-1α

## Abstract

Hesperidin (Hes) protects different organs from damage by acting as a potent antioxidant and anti-inflammatory. This study aims to evaluate the gastroprotective effects of free hesperidin and its chitosan nanoparticles (HNPs) against ethanol-induced gastric ulcers in rats, hypothesizing that HNPs will enhance bioavailability and therapeutic efficacy due to improved solubility and targeted delivery. HNPs were synthesized via ion gelation and characterized using TEM, SEM, and zeta potential analyses. Key assessments included gastric acidity, histological analysis, and markers of inflammation, oxidative stress, and apoptosis. HNPs significantly decreased gastric acidity, reduced inflammatory and apoptotic markers, and enhanced antioxidant enzyme activities compared to free hesperidin and esomeprazole. Furthermore, Sirt-1, PGC-1α, HO-1, and FOXO1 gene expression were also evaluated. HNPs raised Sirt-1, PGC-1α, HO-1, and downregulated FOXO1, and they suppressed the activities of NF-κB p65, COX-2, IL-1β, CD86, FOXO1 P53, and caspase-3 and increased Sirt-1 activity. HNPs treatment notably restored antioxidant enzyme activity, reduced oxidative stress and inflammatory markers, and improved histological outcomes more effectively than free hesperidin and esomeprazole. These results indicate that chitosan nanoparticles significantly enhance the gastroprotective effects of hesperidin against ethanol-induced gastric ulcers, potentially offering a more effective therapeutic strategy. Further research should explore the clinical applications of HNPs in human subjects.

## 1 Introduction

A prevalent illness that affects millions of individuals globally is gastric ulcers (GU). Taking into account the prevalence data worldwide, a novel treatment strategy is required (Lanas and Chan, 2017). Open sores in the upper portion of the digestive system are called ulcers that can cause internal bleeding in addition to discomfort in the stomach. There are two different kinds of ulcers in the stomach: duodenal and gastric. When the biological balance between defensive and aggressive elements in the gastrointestinal system is upset, gastric ulcer disease, a multicausal and complicated illness, develops (Yeo et al., 2018).

Gastric ulcers can result from *Helicobacter pylori*, alcohol, or exposure to stressful conditions (Brown and Wilson, 1999). In addition, drug usage particularly the use of NSAIDs, antibiotics, antipsychotics, and antidepressants is frequently associated with GU (Philpott et al., 2014).

One of the main factors contributing to injury to the stomach mucosa is alcohol (Lanas and Chan, 2017). Alcohol stimulation can cause direct injury to the capillary endothelial cells in the stomach mucosa, which could lead to intragastric bleeding. Previous research has demonstrated that the primary causes of ethanol-induced GU are oxidative stress and inflammatory response (Yeo et al., 2018). Accretion of ROS and increased oxidative stress lead to lipids and protein oxidization consequently mucosal barriers increase the penetrability of the gut, rouse NF-κB signaling inflammatory cytokines generation, and finally cause mucosal apoptosis (Aziz et al., 2019). The symptoms of ulcerative gastritis include inflammation, mucosal ulceration, bleeding, and perforation (Aziz et al., 2019).

However, peptic ulcer traditional medications such as (omeprazole, Esomeprazole, Pantoprazole, Famotidine, and ranitidine) which belong to proton pump inhibitors (Inhibition of the gastric H+/K+-ATPase (proton pump) enzyme system) and H2-receptor blockers (Blocking the action of histamine at the histamine H2 receptors of parietal cells) are just a few examples of pharmaceuticals that are known to have a variety of side effects, including gynecomastia, impotence, arrhythmia, hematopoietic changes, and hypersensitivity (Palle et al., 2018). Moreover, these medications have inadequate ulcer healing, and ulcer recurrence, all of which place a large cost burden on patients and healthcare systems (Newman and Cragg, 2016).

That’s why studies into novel pharmacologically active compounds by screening various plant extracts resulted in the identification of safe and efficient medications with gastroprotective action. Particularly, plants that are the primary source of antioxidants are employed as the herbal reserve to treat ulcer disease (Selmi et al., 2017; Palle et al., 2018). Known as phytochemical components, the capacity of medicinal plants to create a variety of renewable secondary metabolites is what gives them their therapeutic qualities. As a result, many plants have employed these phytochemicals as a defense strategy against different diseases (Sareea Al-Rekaby, 2017). Natural products account for around 35% of newly released medications on the pharmaceutical market. The majority of substances employed in clinical studies are natural products (Newman and Cragg, 2016).

Flavonoids are among the most important types of secondary metabolites in plants (Ghasemzadeh and Jaafar, 2011). Along with having antioxidant properties, flavonoids also have a variety of biological functions that contribute to human health (Elshazly et al., 2018). These include anti-inflammatory, anti-ulcer, anti-viral, anti-cancer, anti-diabetic, and cytotoxic properties (Ghasemzadeh and Jaafar, 2011).

As a flavanone glycoside, hesperidin was first isolated by the French chemist Lebreton from citrus peel. Investigations have shown that hesperidin could protect against acute gastric stress models and ethanol-induced ulcers (Elshazly et al., 2018; da Silva et al., 2019; Ozyigit et al., 2024).

Despite the promising effects of hesperidin, its poor bioavailability necessitates innovative delivery strategies to enhance its therapeutic potential (Ávila-gálvez et al., 2021; Sip et al., 2023). Nanoformulation of hesperidin may enhance its solubility, stability, and bioavailability, thereby maximizing its therapeutic effects against gastric ulcers (Jangde et al., 2022; Bansal et al., 2024).

The pharmacological action of nano-formulated materials has been thoroughly studied during the past 20 years. The manufacture of nanoscale changes a substance’s physical and chemical properties, which may modify a substance’s quantum size, microscopic, solubility, surface charge, and medicinal activity (Khan et al., 2019; Joseph et al., 2023). Nanomaterials have been used in a variety of scientific and technology-related applications, including biomedical applications, as well as environmental cleanup (Arcos, 2023).

Chitosan is the ideal natural polymer, with hemostatic, and varied biological capabilities (antitumoral, antibacterial, antioxidant, and anti-inflammatory) (Jin et al., 2021; Piekarska et al., 2023; Almukainzi et al., 2024). The major amine group, which is located at the C-2 of the glucosamine residues, is the most crucial functional group for the biological activity of chitosan. The deacetylation degree (DDA) and molecular weight (MW) are the two factors with the greatest impact on bioactivities. Chitosan and its derivatives with higher DDA and lower MW were discovered to have greater antibacterial, antioxidant, and anticancer effects (Kim, 2018). Chitosan nanoparticles loaded with hesperidin have been produced for nasal delivery to suppress cytokine Storm Syndrome in a Mouse Model of Acute Lung Injury inflammatory lungs. Chitosan nanoparticles showed superior cellular absorption in the microenvironment of inflammation in comparison to free hesperidin (Jin et al., 2021). Another study was conducted to evaluate Chitosan nanoparticles/hesperidin as an antioxidant and antitumor activities. The study has established that Chitosan nanoparticles/hesperidin, in a highly soluble form, increase antioxidant and antitumor activities in contrast to the poorly soluble form of hesperidin alone (Almukainzi et al., 2024).

Consequently, it is thought that hesperidin and chitosan together might have a synergistic impact on inflammation and oxidative stress (Elmoghayer et al., 2024a; Elmoghayer et al., 2024b; Almukainzi et al., 2024).

This study aims to evaluate the gastroprotective effects of free hesperidin and its chitosan nanoparticles (HNPs) against ethanol-induced gastric ulcers in rats, focusing on the Sirt-1/FOXO1/PGC-1α/HO-1 signaling pathway.

## 2 Materials and methods

### 2.1 Drugs and chemicals

Esomeprazole was purchased from AstraZeneca, Egypt. Hesperidin, Dimethyl sulfoxide (DMSO), and polyethylene glycol (PEG) were purchased from Sigma-Aldrich, United States. Chitosan (poly (D-glucosamine) deacetylated chitin; molecular weight 100–300 K Da (viscosity (20°C): 287.6 cps Deacetylated degree 93%–95%. LANXESS Company, India. Sodium tripolyphosphate (STPP) with a purity of 85% was purchased from LANXESS Company, India. Acetic acid with a purity of 96% (Research-lab fine chem industries, India). Ethanol and sodium hydroxide (NaOH) were purchased from El-Nasr Pharmaceutical Chemicals Co., Cairo, Egypt. Deionized water (Stakpure, Waters, United States). The highest analytical grade was used for every chemical used in this study.

### 2.2 Preparation of hesperidin nanoparticles

The ionic gelation method was used for the preparation of hesperidin/chitosan nanoparticles. Chitosan nanoparticles cross-linked by sodium tripolyphosphate (STPP) containing hesperidin (HNPs) were prepared by ionotropic gelation. Nine grams of chitosan were dispersed in 1,200 mL of 3% acetic acid using magnetic stirrer (Stuart, Caliber Scientific United States) at 200 rpm and 50°C for 30 min, 6 g of drug powder was added (chitosan/hesperidin ratio was 1.5:1 W/W) and stirred for additional 4 h, allowed to equilibrate for 24 h then continue stirring for 1 h, adjust pH to 5 with the aid of 4% NaOH. 300 mL of TPP (1%W/V) was added stepwise with the aid of a 20 mL syringe, continued stirring for an additional 30 min, and the dispersion was allowed to prop sonicator (SONIC VIBRA CELL, United States). Finally, the nanoparticles were separated with the aid of a cooling centrifuge at 10,000 rpm for 10 min at −4°C (Centurion Scientific, United Kingdom). Then, the resulting nanoparticles were washed twice with deionized water and then allowed to freeze until completely dry (Christ Benchtop Freeze dryer, Germany). Drug-free chitosan nanoparticle (CNP) was prepared by the same procedures except for the addition of hesperidin powder (Wrann et al., 2014).

### 2.3 Characterization of hesperidin nanoparticles (HNPs)

#### 2.3.1 Percentage yield, drug entrapment efficiency (DEE), and loading capacity (LC)

The % was evaluated by weighing the total lyophilized powder using an analytical weighing scale (Sartorious, United States). Loading capacity (LC) and entrapment efficiency (DEE) were determined with the indirect method. After centrifuging the HNPs formulation, the amount of medication that was un-entrapped was assessed in the supernatant using a calibration curve. To reduce handling errors, the entire analysis was performed in three duplicates. While DEE is the proportion of the medication that is successfully encapsulated inside the system from the entire drug that was initially added, LC measures the amount of medication that is successfully loaded onto a given mass of CNPs (Huang et al., 2012).

The % yield, DEE, and LC can be calculated according to the following Equations 1–3:

$$\text{The percentage yield}=\left(\text{Total amount of HNPs}\right)/\left(\text{total amount of all ingredients }\left(\text{STPP}+\text{chitosan}+\text{drug}\right)\right)\times 100$$ (1)

$$\%\text{DEE}=\left(\text{total drug conc}.-\text{supernatant drug conc}.\right)/\left(\text{total drug conc}.\times 100\%\right)$$ (2)

$$\%\text{LC}=\left(\text{total amount of drug added}-\text{ the amount of untrapped drug}\right)/\text{total mass of HNPs}\times 100\%$$ (3)

#### 2.3.2 Zeta potential evaluation, polydispersity index (PDI), and average particle size

Particle size determines the efficacy of HNPs, Zeta potential, and PDI are measures of colloidal stability and homogeneity, respectively. Zeta Sizer Nano (Malvern Analytical Ltd., United Kingdom) was used for assessing particle size, PDI as well as zeta potential (Chu et al., 2020).

#### 2.3.3 Scanning electron microscope (SEM) and transmission electron microscope (TEM)

The morphology and surface properties of HNPs and pure hesperidin were assessed using Scanning electron microscope (SEM) examinations. The lyophilized powder was suspended in alcohol and sonicated then, one drop of the suspension was allowed to be spread over a glass slide and allowed for complete drying then transmitted over the metal stub’s top (cupper) on a silicon electro-conductive chip piece. Using a 10 kV electron acceleration (JEOL, JSM-6510LV, Japan) of various magnifications, the materials were coated with gold for 1 minute on the stubs.

The sample was mounted on a carbon-coated grid, then the sample was air-dried and photographed with a transmission electron microscope (TEM, JEM-2100F electron microscope, JEOL Ltd., Tokyo, Japan).

#### 2.3.4 Thermal stability (DSC), X-ray diffraction (XRD) analysis, and fourier transform infrared spectroscopy (FTIR) analysis

The differential scanning calorimetry was performed on TA instruments-Waters LIC, United States, to examine the thermal behavior of samples of raw materials, drug-free nanoparticles (CNPs), and drug-loaded nanoparticles (HNPs). The heat flow was 10°C/m, each sample was carefully weighed (1.5–4 mg) using a microbalance (Sartorius, Germany), and the samples were heated from 50°C to 350°C. The reference was an empty aluminum pan.

The X-ray diffraction of HNPs and pure hesperidin was carried out. Diffractograms according to Bragg’s law were acquired using an XRD diffractometer (APD2000 pro, GNR, Italy, software CRYSTAL IMPACT, Bonn, Germany) of CuK radiation, 35 kV voltage monochromatic with electric current (25 mA). 4.95°–79.75° was the range for the 2 diffraction angles. The FTIR was performed to determine the interactions of the components of the formulation, emphasizing the stability of the suggested system. Pure hesperidin powder and HNPs were subjected to FTIR spectroscopic analysis using BRUKER (United States) (Banni et al., 2010).

#### 2.3.5 Drug release *in-vitro* study

The dissolving characteristics of HNPs and pure hesperidin powder were compared. Type II dissolution equipment (Copley Scientific, United Kingdom) was used for the studies. The temperature was 37°C ± 5°C while the paddles rotated at 75 rpm. 900 mL of 0.1 N HCl as the dissolution medium. The dissolving medium was filled with precisely weighed samples containing the equivalent of 10 mg of hesperidin. A 0.2 µm syringe filter was used to filter samples of the dissolution medium (3 mL), which were then taken out at various time intervals for spectrophotometric analysis at 283 nm. Withdrawn samples were made up for with a new medium. The dissolution tests were carried out three times, samples were collected at 5, 10, 15, 30, 60, 90, and 120 min.

### 2.4 Animals and ethical approval

The research was authorized by Tanta University’s Faculty of Pharmacy’s Research Ethics Committee and adhered to CIOMS’s (International Organizations Council for Medical Sciences) standards (Code of Protocol: TP/RE/6/23p-0036). All methods were reported in accordance with ARRIVE 2.0 guidelines. All precautions were taken in order to minimize animal suffering during the experiments. Rats were given isoflurane anesthesia before being cervical dislocated.

### 2.5 Experimental design

Overall 54 mature male albino rats (180–220 g) were bought from the animal house of Cairo’s National Research Center. Nine groups of animals, each including six rats, were used: (I) Control vehicle group: rats were given (normal saline/DMSO/PEG) vehicle by oral gavage for 14 days. (II) Control polymer group: rats were given chitosan polymer by oral gavage for 14 days. (III) Esomeprazole group (Esmo): a positive control group receiving Esmo 20 mg/kg body weight dissolved in 0.5 mL normal saline by oral gavage, once daily for 14 days (Sabiu et al., 2016). (IV) Hesperidin group (Hes): rats were given Hes 100 mg/kg body weight dissolved in 0.5 mL vehicle by oral gavage, once daily for 14 days (Elshazly et al., 2018). (V) Nano Hesperidin group (Nano Hes): rats were given Nano Hes 100 mg/kg body weight dissolved in 0.5 mL vehicle by oral gavage, once daily for 14 days. (VI) ethanol group (ETOH): rats were given 0.5 mL vehicle by oral gavage for 14 days then on day 14, rats were given just one dose of ethanol (5 mL/kg), which was administered through oral gavage (Arab et al., 2015). (VII) ETOH + Esmo: Rats were pretreated with Esmo for 14 days followed by a single injection of ethanol on day 14. (VIII) ETOH + Hes: Rats were pretreated with free Hes for 14 days, then after that a single injection of ethanol on day 14. (IX) ETOH + Nano Hes: Rats were pretreated with Nano Hes for 14 days followed by a single injection of ethanol on day 14. The animals were euthanized under anesthesia 1 h following ethanol administration, also, their stomachs were gathered.

### 2.6 Induction of gastric ulcer

The acute gastric mucosal injury was induced using a single intragastric dose of absolute ethanol (5 mL/kg) that was administered via orogastric intubation, following 24 h of fasting without anesthesia, as previously described (Koukaras et al., 2012). The control group received the same volume of vehicle instead of ethanol.

### 2.7 Sample collection

One hour after receiving ethanol, rats were given isoflurane anesthesia before being cervical dislocated and euthanized. From the greater curvature, stomachs were removed and opened. Tubes were used to collect gastric fluid, and ordinary saline was used to clean the stomach tissues. Then, randomly chosen stomachs from each group were examined for histopathology and immunohistochemistry. For use in the various assays listed below, The remaining tissue was weighted and stored at −80°C after being sliced into bits.

### 2.8 Measuring the pH of the stomach

Gastric juice was extracted from opened stomachs through the larger curvature, 1 mL of distilled water was added after being centrifuged at 3,000 rpm for 10 min at 4°C and its pH was determined with a pH meter (Bucharest, Romania) (Yuan et al., 2006).

### 2.9 Gastric juice acidity assessment

The addition of an equal volume (I ml) of water and centrifuged gastric content in a conical flask (50 mL) to quantify the total acidic content was done following published procedures. In the flask, drops of phenolphthalein indicator were then added, and subjected to titration by using NaOH until a pink color developed. The volume of titer (NaOH 0.01 N) used had been assessed (Raish et al., 2018).

### 2.10 Determination of oxidative stress

All samples from animals were biochemically analyzed under previously published investigations. Extracted tissues of the stomach were kept at −80°C, according to data from recent studies. PBS buffer (500 mL) was used to homogenize the prepared tissue before centrifugation. Using various commercial kits of ELISA, the supernatants of stomach homogenate were submitted to assay oxidative stress markers malondialdehyde (MDA) (MyBioSource. Co, Cat No. MBS268427) and Nitric Oxide (NO) (MyBioSource. Co, Cat. No. MBS723386) using the manufacturer’s instructions. The intensity of the obtained color was measured at 450 nm.

### 2.11 Estimation of antioxidant enzyme activities

Using kits of ELISA, the supernatants of stomach homogenate were submitted to assay antioxidant enzyme activities superoxide dismutase (SOD) (CUSABIO. Co, Cat No. CSB-E08555r) and catalase activity (CAT) (MyBiosource. Co Cat No. MBS2600683) using the manufacturer’s instructions. At 450 nm, the intensity of produced color was measured.

### 2.12 Estimation of myeloperoxidase (MPO) enzyme activity in gastric tissue

MPO ELISA kit, CUSABIO, Co, Cat No. CSB-E08722r was employed for the estimation of MPO activity as a marker of neutrophil infiltration, according to the manufacturer’s instructions at 450 nm.

### 2.13 Estimation of inflammatory cytokines

To estimate the concentrations of various proinflammatory biomarkers in the homogenate isolated from rat stomach tissue, commercially available ELISA kits were used according to the manufacturer’s instructions.

Pro-inflammatory mediators such as TNF-α (CUSABIO. Co, Cat No. CSB-E11987), HO-1 (CUSABIO. Co, Cat No. CSB-E08267r), and anti-inflammatory marker (IL-10) (MyBiosource, Cat No. MBS269138) were measured at 450 nm.

### 2.14 Estimation of M1 macrophages CD38

CD38 ELISA kit, CUSABIO, Co, Cat No. CSB-EL004929RA was employed for the estimation of CD38 activity as a marker of M1 macrophages, according to the manufacturer’s instructions at 450 nm.

### 2.15 Quantitative real-time PCR (qRT-PCR) of Sirt-1, PGC-1α, FOXO-1, and HO-1

Easy-spinTM total RNA extraction kit was utilized, and after that, RNA total was extracted (iNtRON Bio, Inc., Korea). The analyses were carried out utilizing qRT-PCR, and a quantiTect reverse transcription kit was obtained (QIAGEN, Hilden, Germany). Temperatures (50°C–99°C) were used for melting curve analysis. The initial step was maintained at 95°C/10 s along with 55 repetitions of 15 seconds of annealing at 60°C.

The following sequences of primers had been utilised: Sirt-1 (forward primer: 5′- CAC-CAG-AAA-GAA-CTT-CAC-CAC-CAG -3′, reverse primer: 5′- ACC-ATC-AAG-CCG-CCT-ACT-AAT-CTG -3′) (Braidy et al., 2015), PGC-1α (Forward primer: 5′- AAT​GAA​TGC​AGC​GGT​CTT​AG-3′, reverse primer: 5′- GTC​TTT​GTG​GCT​TTT​GCT​GT-3′) (Wrann et al., 2014), FOXO-1 (Forward primer: 5′- GCTGCATCCATGGACAACAACA-3′,reverse primer: 5′- CGA​GGG​CGA​AAT​GTA​CTC​CAG​TT-3′) (Huang et al., 2012). HO-1 (Forward primer: 5′- GGC​TTT​AAG​CTG​GTG​ATG​GC-3′), reverse primer: 5′- GGG​TTC​TGC​TTG​TTT​CGC​TC-3′) (Chu et al., 2020), and β-actin (forward primer: 5′-TCC​TCC​TGA​GCG​CAA​GTA​CTC​T-3′, reverse primer:5′- GCT​CAG​TAA​CAG​TCC​GCC​TAG​AA-3′) (Andrews et al., 2002). The ΔCt value was computed with the specified software.

The Ct value expresses the cycle number when the fluorescence curve reaches the baseline value. Relative mRNA expression of target genes was calculated using fold changes methods (2^−ΔΔCt^) (Yuan et al., 2006).

### 2.16 Gross observation of gastric tissue injury

After collecting the stomach contents, the stomachs of all experimental groups were opened along the greater curvature. They were then washed with normal saline to remove debris, and pinned for ulcer scoring. Gastric mucosal damage was evaluated using a scoring system based on criteria from Haule et al. (2012) and Sadek (2022). The scores range from 0 (no pathological changes) to 5 (perforated ulcer), with intermediate scores reflecting varying degrees of damage such as hyperemia, hemorrhagic spots, and ulcers of different sizes.

### 2.17 Histopathological assessment of gastric tissues

Histopathology for histological and mucosal evaluations, Gastric tissues were stained with hematoxylin and eosin (H and E), Periodic acid Schiff (PAS) stain solution, viewed under a light microscope after being fixed in formalin buffer 10%, immersed in paraffin, and sliced into 5-m slices (Raish et al., 2018) using histopathology scoring system: Intact mucosal epithelium (Grade 0), Superficial lesion involving mucosa only (Grade 1), Deeper lesion involving mucosa and submucosa (Grade 2), Deep lesion involving mucosa, submucosa extending to the tunica muscularis (Grade 3) (Andrews et al., 2002).

### 2.18 IHC (immunohistochemical analysis)

The immunohistochemical staining procedure was used according to Saber et al. (2019). The avidin-biotin-peroxidase method was used to immune-stain sections from stomach paraffin blocks for NF-κB p65, IL-1β, Cox-2, caspase-3, P53, Sirt-1, FOXO1, as well as CD86 by the method of avidin-biotin-peroxidase. Before microwave treatment (0.01 M Trisodium citrate), Gastric tissue was cut into paraffin slices, and dewaxed in xylene. Then, rehydrated in descending alcohol concentrations for antigen retrieval. To deactivate endogenous peroxidase, sections were incubated in 10% H_2_O_2_ before being treated with primary antibodies (rat monoclonal antibodies) NF-κB p65, IL-1β, Cox-2, caspase-3, P53, Sirt-1, FOXO1, and CD86.

### 2.19 Statistical analyses

Data were expressed as mean values ±standard deviation (mean ± S.D). The Kolmogorov-Smirnov normality test was applied to verify the data normality. The differences between groups were determined with one-way ANOVA followed by Tukey’s multiple comparisons. A *p*-value of <0.05 was used for statistical significance establishment. For statistical calculations, GraphPad Prism, version 5 (GraphPad Software Inc., La Jolla, CA, United States) was utilized.

## 3 Results

### 3.1 Entrapment efficiency, and loading capacity

The chitosan/STPP nanoparticles’ entrapment efficiency was 88% while loading capacity (LC) was 29.33% (Table 1).

**TABLE 1:** Polymeric HNPs characterization.

Parameter	Mean ± SD	Range
%EE	88.0 ± 4.25	83.86 to 91.7
% LC	29.33 ± 3.6	25.58 to 32.75
Particle size (nm)	358.03 ± 5.72	128.7 to 487.5
PDI	0.408 ± 0.020	0.39 to 0.43
Zeta potential (mV)	23.92 ± 0.38	23.5 to 24.24

### 3.2 Particle size, PDI as well as zeta potential analysis

**FIGURE 1:**
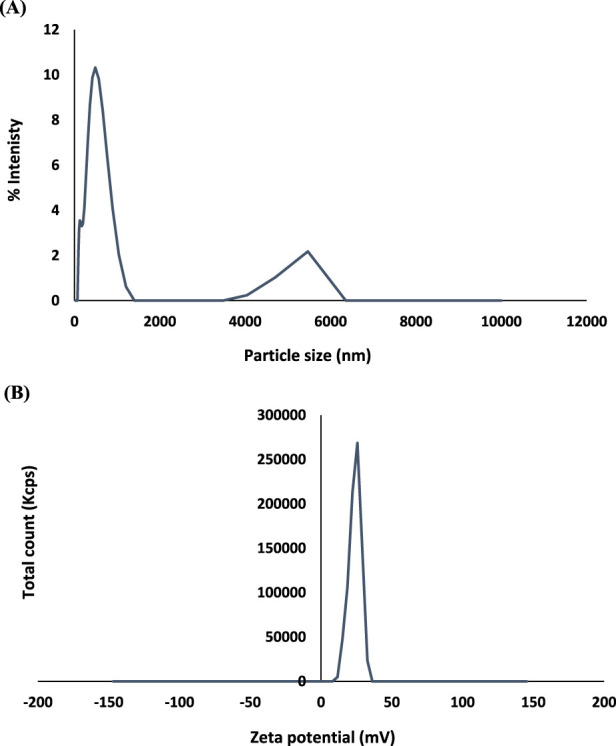
**(A)** Particle size (nm), and **(B)** Zeta Potential (mv) of HNPs.

The results of particle size showed The mean particle sizes of the produced HNPs in this study were 358.03 ± 5.71 nm. The PDI of HNPs was 0.408 ± 0.020. The produced chitosan nanoparticles’ zeta potential was 23.91 ± 0.38 mV (Figure 1; Table 1).

### 3.3 Scanning electron microscope (SEM) and transmission electron microscope (SEM)

**FIGURE 2:**
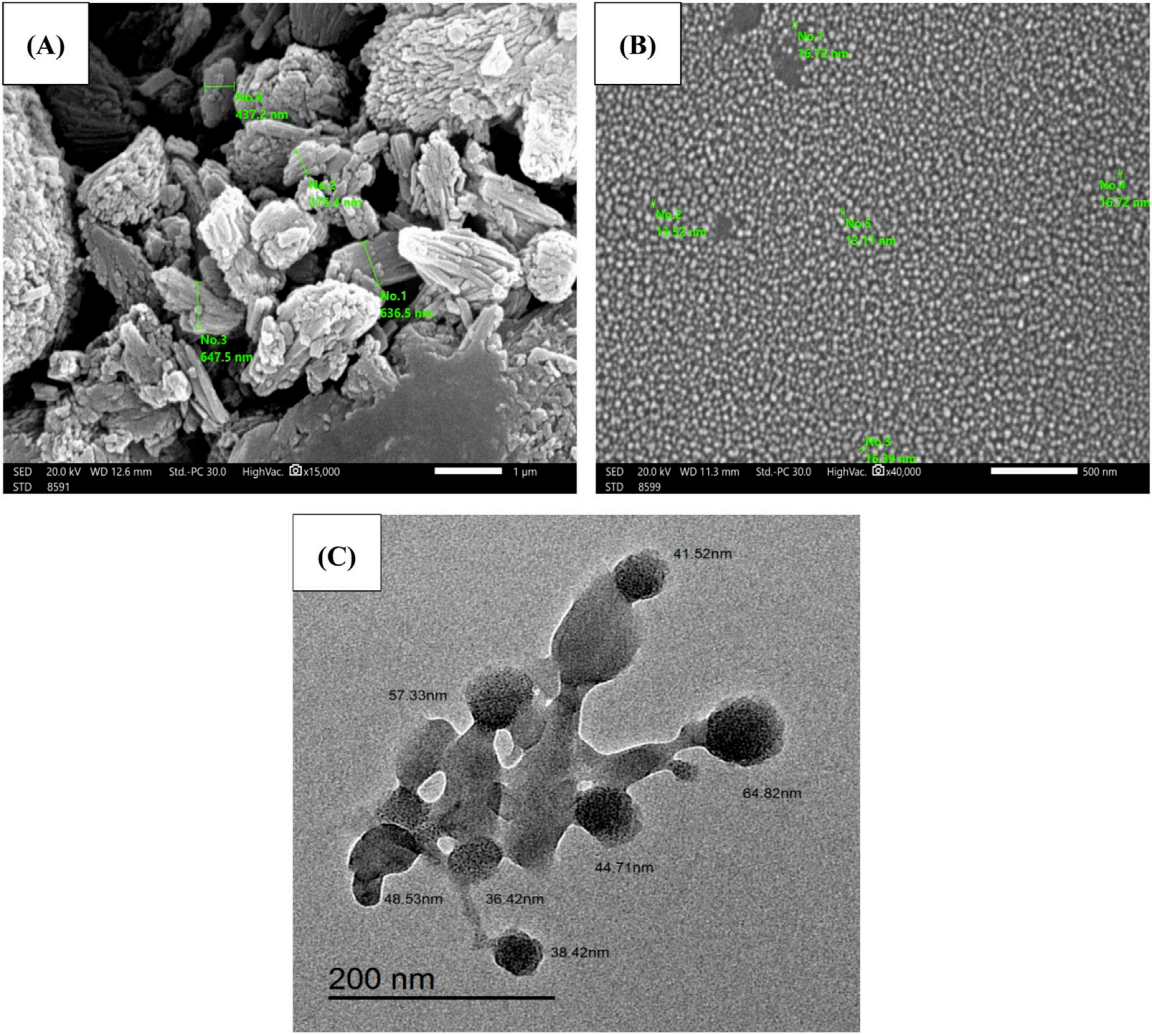
**(A)** SEM of free Hes, **(B)** HNPs (400,00x), and **(C)** TEM of HNPs.

As illustrated in Figure 2, free Hes and HNPs morphology, size, and shape were assessed by SEM. Free Hes (Figure 2A) appeared as aggregated particles with uneven distribution (magnification power 15,000), on the other hand, HNPs (Figure 2B) displayed well-separated, smooth, spherical particles with particle size less than 20 nm (magnification power 40,000). The TEM illustrated the cross-linked chitosan polymer (cluster shape) entrapping small spheres of hesperidin (dark spheres) (Figure 2C).

### 3.4 Thermal stability (DSC)

**FIGURE 3:**
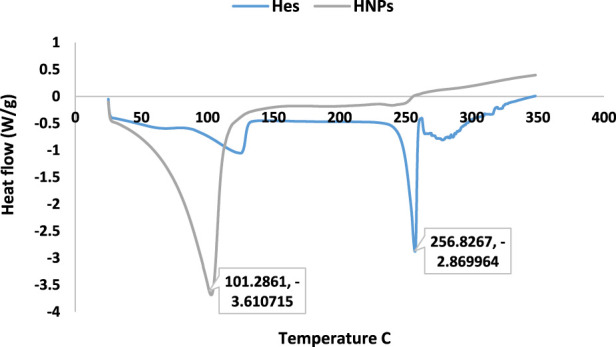
DSC of free Hes, HNPs, and CNPs.

The results of DSC thermograms (heat flow 10°C/min) were summarized in Figure 3, Table 2. The results explicated the characteristic melting point of Hes ( $T_{m}^{{}^{\circ}}$ 256.89°C, 2.874 J/g) (Elmoghayer et al., 2024b).

**TABLE 2:** Thermal stability of hesperidin, CNPs, and HNPs.

	Temperature (°C)	Peak intensity (J/g)
Hes	257.01 (melting point)	−2.87
CNPs	82.29 (dehydration peak)	−2.26
HNPs	102.97 (dehydration peak)	3.69

On the other hand, the DSC of CNPs showed the characteristic endothermic peak (dehydration temperature 77.02°C). The exothermic peak is related to the thermal degradation of the chitosan polymer (Gharanjig et al., 2020).

Concerning the DSC of HNPs the characteristic endothermic peak disappeared completely in the thermogram of HNPs indicating the complete encapsulation of hesperidin inside the chitosan polymer, while another broad peak appeared ( $T_{m}^{{}^{\circ}}$ 102.7°C, −3.68 J/g). The new endothermic peak could be attributed to the incorporation of some water molecules due to the nature of chitosan with a high affinity to hydration. The exothermic peak related to chitosan degradation disappeared also indicating higher stability of the nanoparticle. The DSC of HNPs appeared as if it is an entirely new compound that differs from its constituents, indicating some type of interaction between Hes and Cs (Gharanjig et al., 2020).

### 3.5 X-ray diffraction analysis (XRD)

**FIGURE 4:**
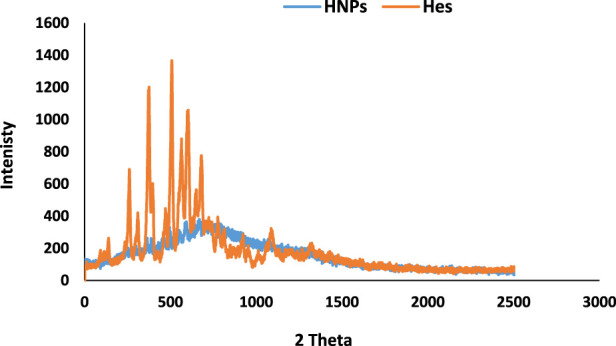
XRD diffractograms of free Hes and HNPs.

Figure 4 showed the suppression of all peaks in diffractograms of HNPs concerning pure drug which showed characteristic sharp peaks at (12.77, 14.24, 16.1, 20.18, 21.89, 22.91, and 25.43°).

### 3.6 Fourier transform infrared spectroscopy (FTIR) analysis

**FIGURE 5:**
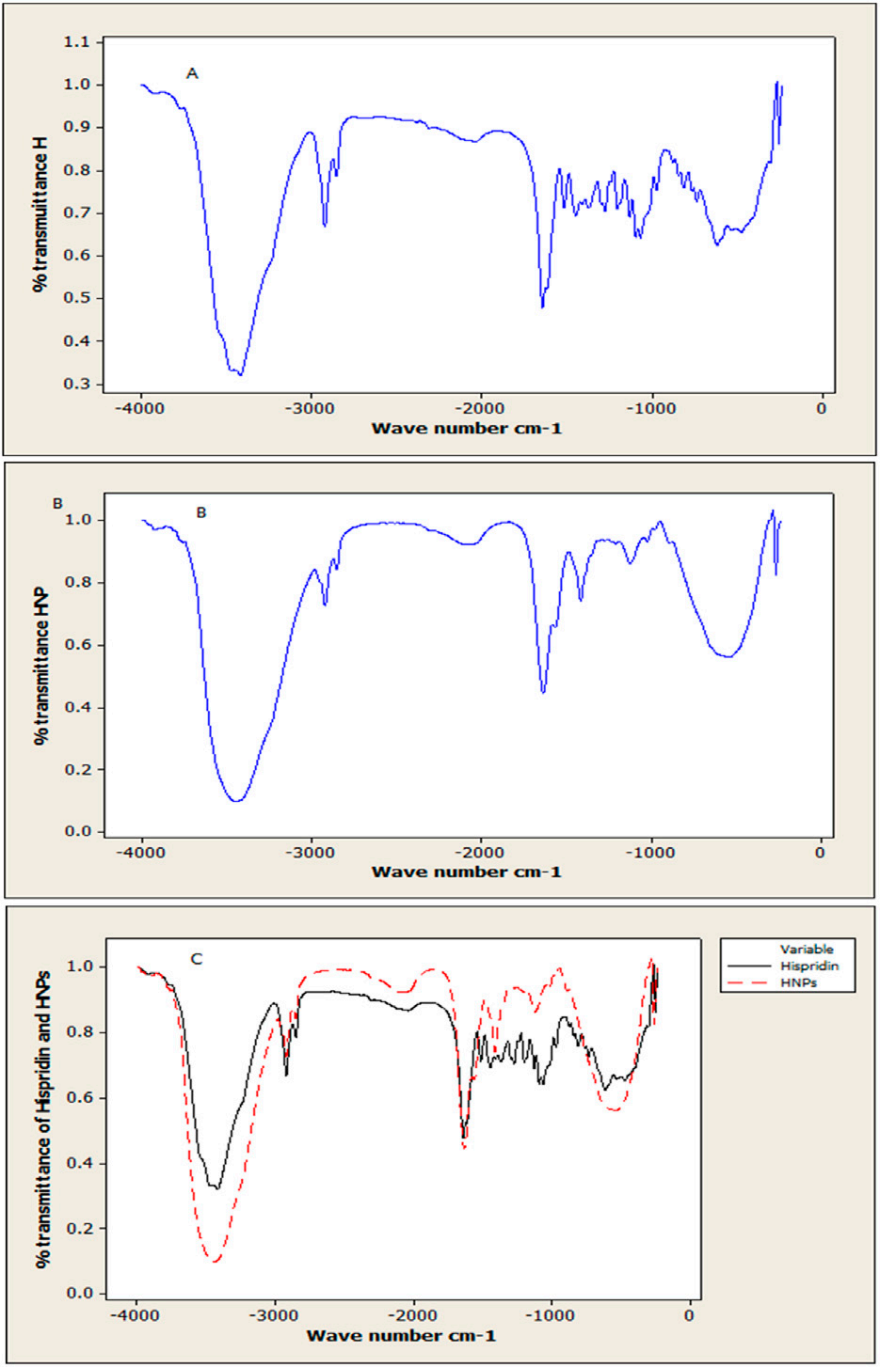
Analysis using FTIR spectroscopy: **(A)** Free Hes **(B)** Modified nano hesperidin loaded in cross-linked chitosan HNPs and **(C)** The overlay of pure hesperidin and HNPs.

Results obtained of free hesperidin and constructed HNPs are illustrated in Figures 5A–C. The spectrums of pure hesperidin (Figure 5A) showed a characteristic at 3,444.77 which, which stands for the hydroxyl group (OH) stretching vibration. Another peak at 2,927.86 represents Alkan CH stretching. On the other hand, at 1,649.09 a distinctive sharp strong peak stands for the carbonyl group (C = O). While the peak at 1,097.47 is specific for C-O aromatic stretching. Otherwise, the FTIR spectrum of HNPs (Figure 5B) showed a slight variation corresponding peak at 3,506 with higher intensity in comparison to pure hesperidin. The peak at 1,147.6 which is responsible for the C-O aromatic stretch became less prominent.

### 3.7 *In vitro* drug release study

**FIGURE 6:**
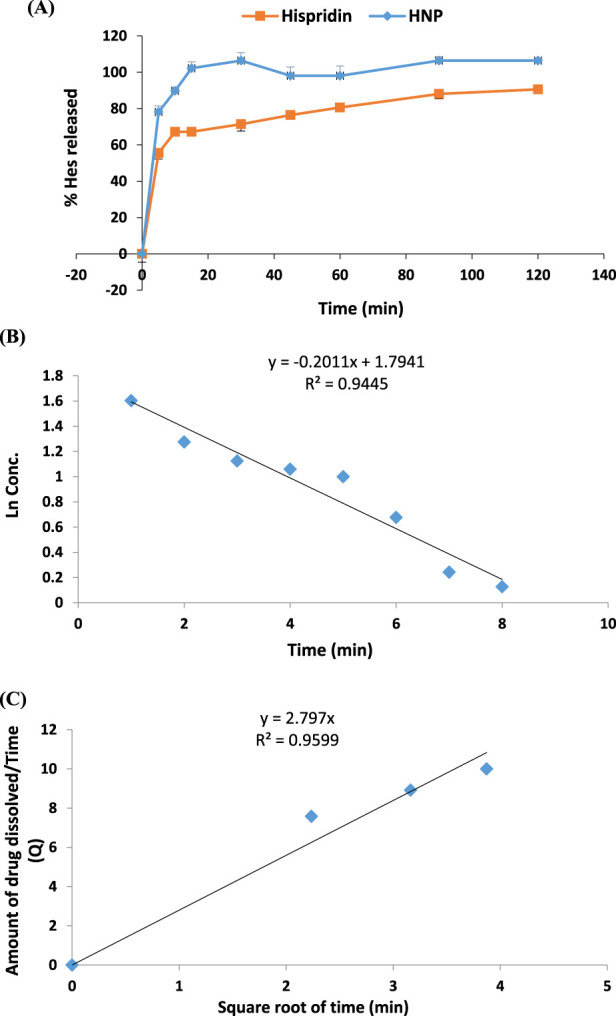
**(A)** Percentage of Dissolved Hesperidin from pure hesperidin powder and HNPs **(B)** Dissolution behavior of Hesperidin powder (first order) **(C)** Dissolution behavior of HNPs (Higuchi).

Emphasized superior solubility of HNPs over hesperidin as shown in (Figure 6A). The dissolving parameters, which were calculated and displayed in Table 3, included the percentage of drug dissolved after 15 and 60 min (Q15% and Q60%) for hesperidin and HNPS. Concerning hesperidin, the Q15% and Q60% were 69.19% and 80.3%, while HNPs showed complete dissolution after 15 min (Q15% = 102.5%). It was clear that pure hesperidin powder dissolution behavior follows the first order kinetic meaning that exponential decrease of the amount dissolved over time and the Equation 4 that describes the dissolution behavior is:

Ln C=Ln C0 –KT (4)

**TABLE 3:** The percentage drug dissolved and % dissolution efficiency of hesperidin from plain powder, HNPs in 0.1 N HCL at 37°C ± 0.5°C.

	Q15% (mean ± SD, n = 3)	Q60% (mean ± SD, n = 3)	$R^{2}$	Drug release behavior
Hesperidin	69.19 ± 1.7	80.31 ± 1.3	0.93.8	First order
HNPs	100.03 ± 2.1	100.86 ± 4.8	0.99	Higuchi

Where C_0 is the initial amount of hesperidin (10 mg), C is the amount of un-dissolved hesperidin, T is the time interval and K is the first order release constant which was 0.2011 (Table 3; Figure 6B). On the other hand, the dissolution behavior of HNPs follows the Higuchi Equation 5 which describes the drug release from the matrix system and the equation is:

$$0.01196\;Q={\text{KT}}^{\wedge }\left(1/2\right)$$ (5)

Where, Q is the amount of hesperidin released at time T, K (=2.797) is the Higuchi release constant and T is the time (Table 3; Figure 6C) (Wójcik-Pastuszka et al., 2019; Shafiei et al., 2021).

### 3.8 Effect of hesperidin (free and encapsulated) on gastric pH and gastric juice acidity

**FIGURE 7:**
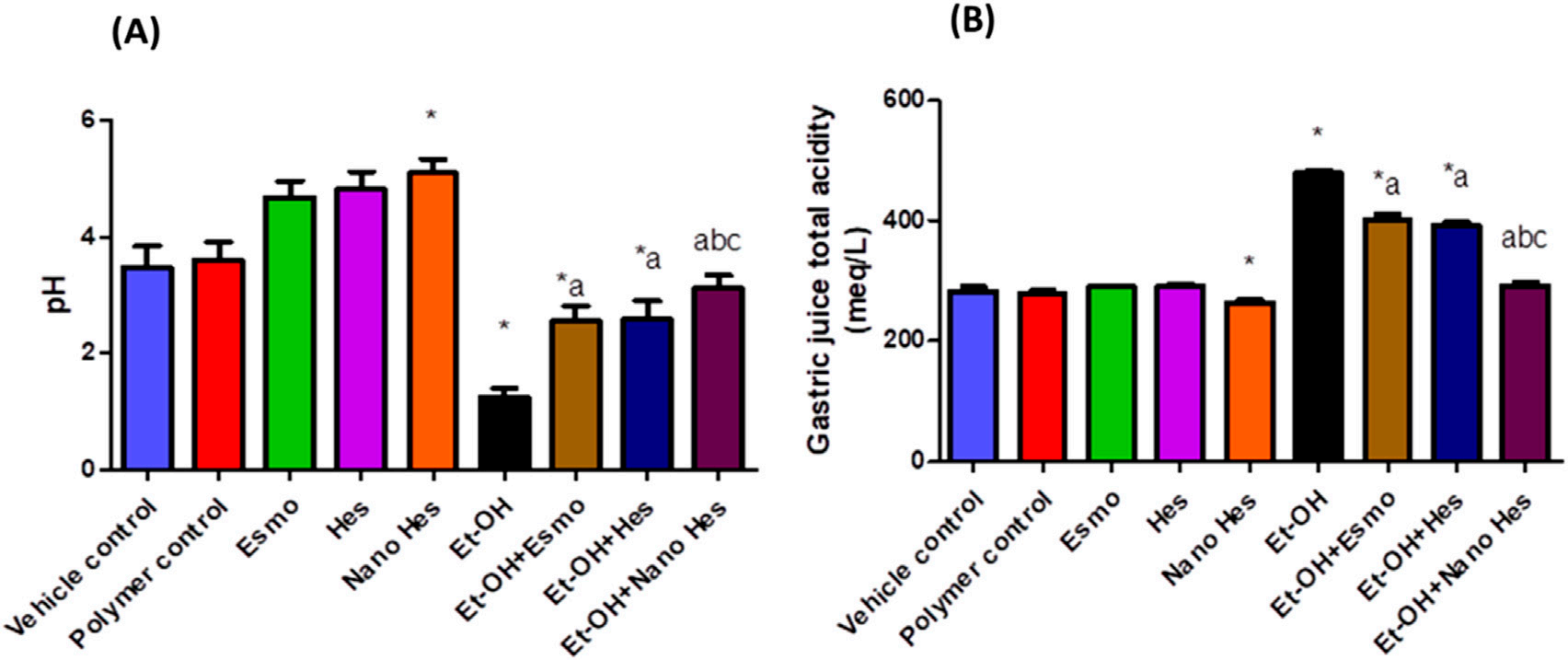
Effect of hesperidin (free and encapsulated) pretreatment on **(A)** gastric pH and **(B)** gastric juice acidity in rats’ ethanol-induced gastric ulcers. Data expressed as mean ± SD (*n* = 6).^*^Relative to vehicle control, ^a^Relative to ethanol-induced ulcerated rats group, ^b^Compared to Esmo ulcerated group, and ^c^Compared to free Hes ulcerated groups. Each group differed significantly from the others at p ≤ 0.05.

As shown in Figures 7A, B significant increase in total acidity (69.36%), and a significant reduction (64.27%) in the stomach juice’s pH were noted in rats’ ethanol-induced ulcers compared with the control vehicle. Pretreatment with Esmo, free Hes, and Nano Hes showed a significant decrease in total acidity (15.96%, 18.15%, 39.02%, respectively), and a significant increase (106.09%, 107.54%, and 150.84%, respectively) in the stomach juice’s pH were observed as compared to the ethanol-induced ulcerated rats. Pretreatment with Nano Hes showed a significant reduction (27.43% and 25.49%, respectively) in total acidity and a significant increase (21.71% and 20.86%, respectively) in gastric juice pH as compared with Esmo and free Hes ulcerated groups.

### 3.9 Effect of hesperidin (free and encapsulated) on gastric oxidative stress (lipid peroxidation and nitric oxide) and antioxidant enzymes (SOD and catalase) content

**FIGURE 8:**
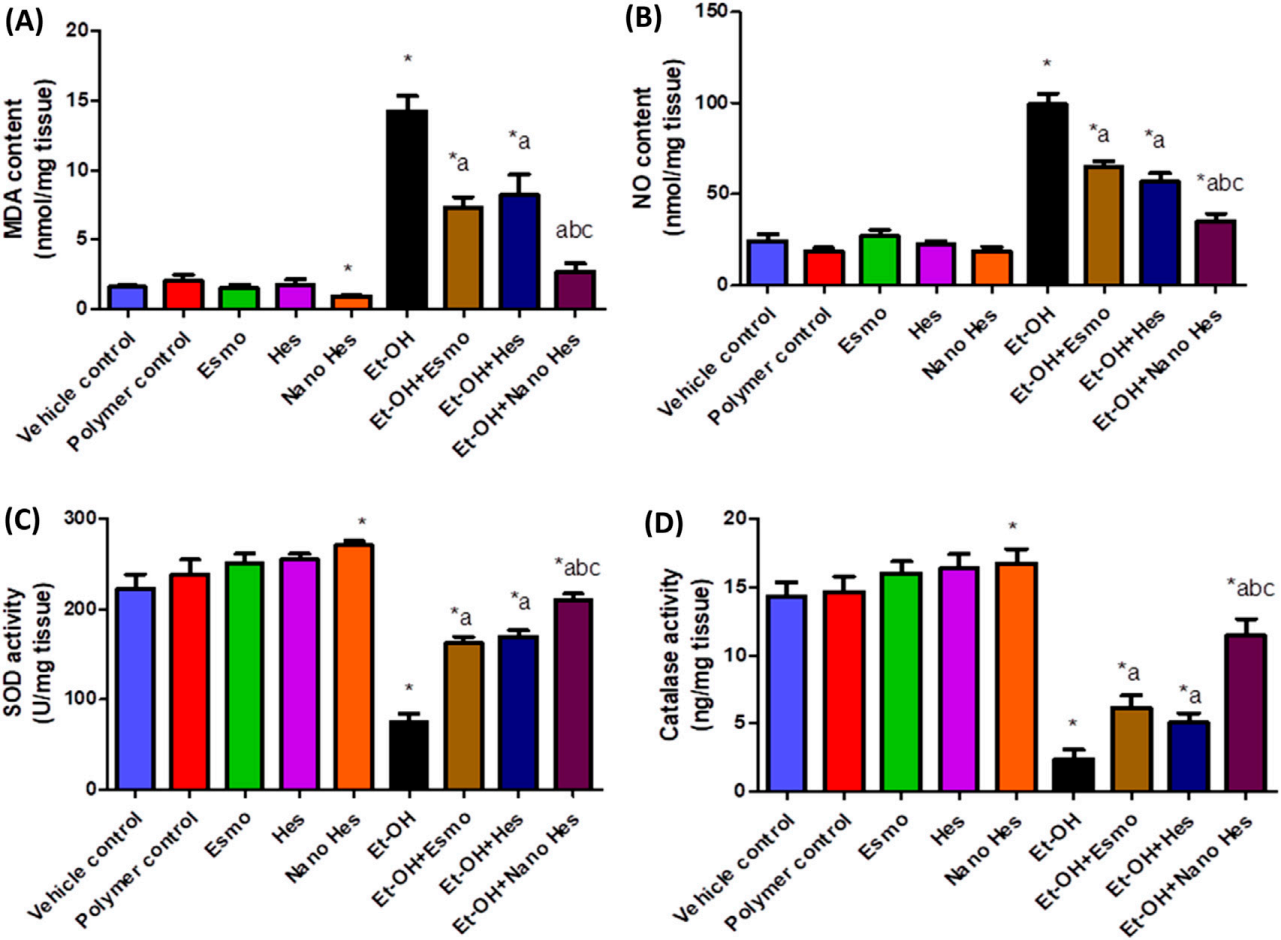
Effect of hesperidin (free and encapsulated) pretreatment on gastric **(A)** MDA, **(B)** NO, **(C)** SOD, and **(D)** Catalase content in rats’ ethanol-induced gastric ulcers. Data expressed as mean ± SD (*n* = 6). ^*^Relative to a vehicle control group, ^a^Compared to the ethanol-induced ulcerated rats’ group, ^b^Compared to Esmo ulcerated group, and ^c^Compared to free Hes ulcerated groups. Each group differed significantly from the others at p ≤ 0.05.

As shown in Figure 8A, Oral administration of ethanol significantly raised MDA content by 797.42% compared with the vehicle control group. Pretreatment with Esmo, free Hes, and Nano Hes significantly decreased MDA content by (48.67%, 42.59%, and 81.02%, respectively) as compared to the ethanol-induced ulcerated rats. It was also found that pretreatment with Nano Hes showed a non-significant change in the content of MDA compared with the polymer control group. Pretreatment with Nano Hes showed a significant reduction (63.02%, and 66.94%, respectively) of MDA content as compared with Esmo and free Hes ulcerated groups (Figure 8A).

Oral administration of ethanol significantly increased NO content by 306.62% compared with the vehicle control group (Figure 8B). Pretreatment with Esmo, free Hes, and Nano Hes significantly decreased NO content by (34.82%, 42.7%, and 64.67%, respectively) as compared to the ethanol-induced ulcerated rats. It was also found that pretreatment with Nano Hes showed a significant reduction (87.11%) in NO content compared with the polymer control. Pretreatment with Nano Hes showed a significant reduction (45.79%, and 38.33%, respectively) of NO content as compared with Esmo and free Hes ulcerated groups (Figure 8B).

Defense antioxidant enzymes like catalase and SOD activity were significantly diminished by (38.46% and 66.16%, respectively) in ethanol-induced ulcerated rats relative to a vehicle control group. Esmo, free Hes, and Nano Hes Pre-treatments recovered this diminished activity by approximately (158.31%, 114.25%, and 382.29%, respectively) for catalase activity and (115.33%, 125.27%, and 178.97%, respectively) for SOD activity as compared to ethanol-induced ulcerated rats (Figures 8C, D, respectively). It was also found that pretreatment with Nano Hes showed a significant reduction (22.02%, and 11.63%, respectively) in catalase and SOD activity, respectively, as compared to the polymer control group. Pretreatment with Nano Hes showed a significant increase (86.71%, and 125.11%, respectively) in catalase activity and a significant increase (29.56% and 23.84%, respectively) in SOD activity as compared with Esmo and free Hes ulcerated groups rats (Figures 8C, D, respectively).

### 3.10 Effect of hesperidin (free and encapsulated) on gastric TNF-α, HO-1 content, and MPO activity

**FIGURE 9:**
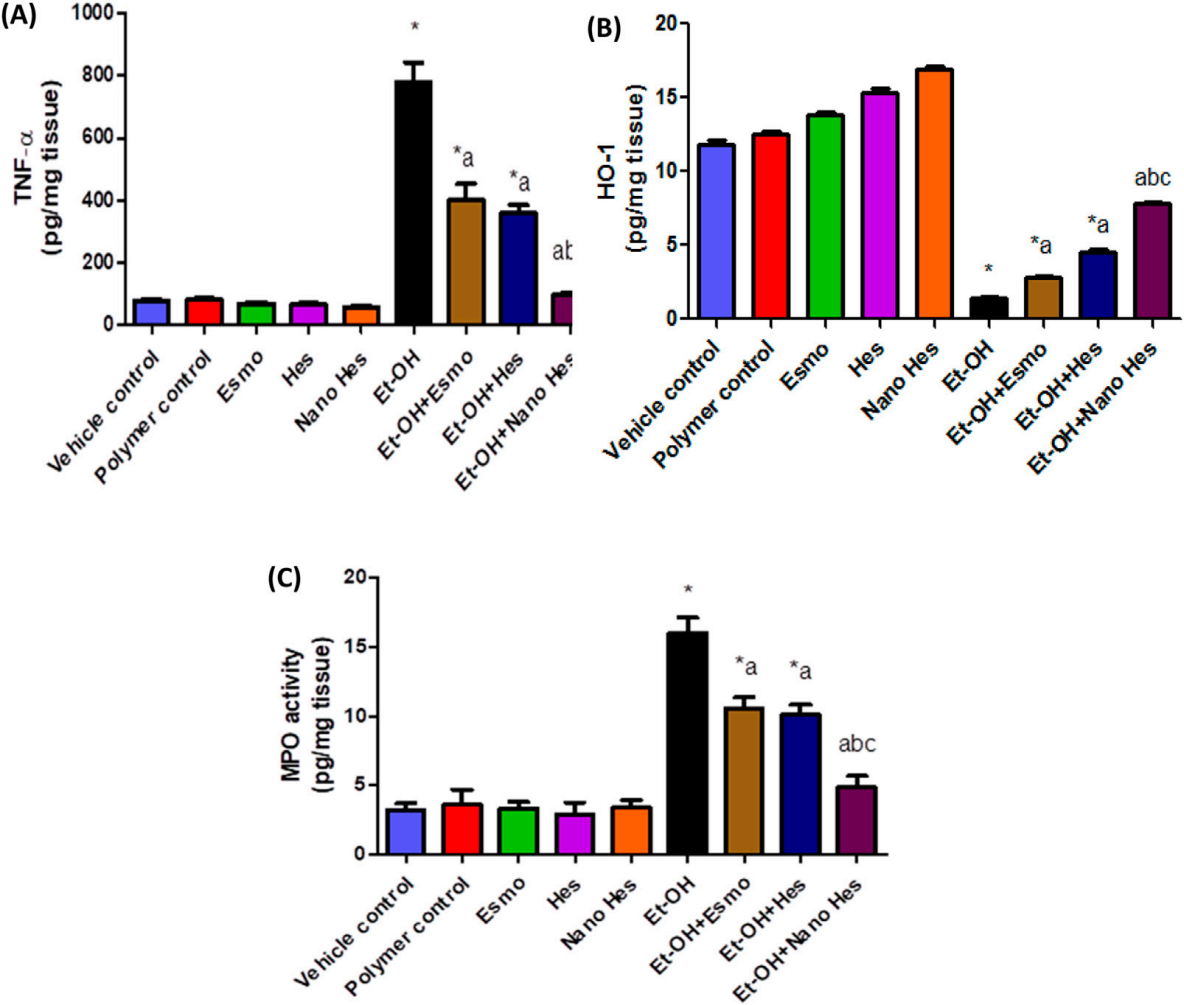
Effect of hesperidin (free and encapsulated) pretreatment on gastric **(A)** TNF- α, **(B)** HO-1 content, and **(C)** MPO enzyme activity in rats’ ethanol-induced gastric ulcers. Data expressed as mean ± SD (*n* = 6/group). ^*^Relative to vehicle control, ^a^Relative to rats’ ethanol-induced gastric ulcers, ^b^Compared with Esmo ulcerated group, and ^c^Compared to free Hes ulcerated groups. Each group differed significantly from the others at p ≤ 0.05.

Oral administration of ethanol significantly elevated TNF-α content by 898.34% as compared to the vehicle control group. Pretreatment with Esmo, free Hes, and Nano Hes significantly decreased TNF- α content by (48.83%, 54.02%, and 87.24%, respectively) as compared to the ethanol-induced ulcerated rats. It was also found that pretreatment with Nano Hes showed a non-significant change in TNF-α content relative to the control polymer. Nano Hes Pretreatment showed a significant reduction (75.07% and 72.26%, respectively) of TNF-α content as compared with Esmo and free Hes ulcerated groups (Figure 9A).

In addition, the administration of ethanol significantly decreased HO-1 content by 87.82% as compared to the vehicle control group. Pretreatment with Esmo, free Hes, and Nano Hes significantly increased HO-1 content by (94.46%, 212.80%, and 438.40%, respectively) as compared to the ethanol-induced ulcerated rats. It was also found that pretreatment with Nano Hes showed a non-significant change in HO-1 content relative to the control polymer. Nano Hes Pretreatment showed a significant reduction (176.86% and 72.12%, respectively) of TNF-α content as compared with Esmo and free Hes ulcerated groups (Figure 9B).

Also, Figure 9C showed the activity of myeloperoxidase (MPO) was significantly increased by 400.94% compared with a vehicle control group. Moreover, Significant mitigation (34.37%, 37.18%, and 69.69%, respectively) of the enhanced neutrophil influx was achieved by pretreatment with Esmo, free Hes, and Nano Hes, respectively. Also, Pretreatment with Nano Hes showed a significant reduction (53.82%, and 51.76%, respectively) of MPO activity as compared with Esmo and free Hes ulcerated groups.

### 3.11 Effect of hesperidin (free and encapsulated) on gastric anti-inflammatory cytokine IL-10

**FIGURE 10:**
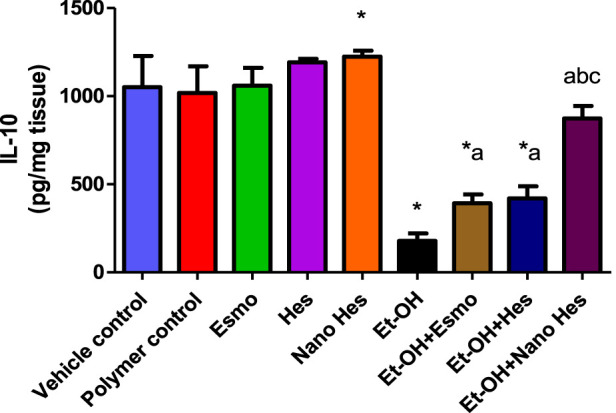
Effect of hesperidin (free and encapsulated) pretreatment on gastric IL-10 content in rats’ ethanol-induced gastric ulcers. Data expressed as mean ± SD (*n* = 6/group). ^*^Compared to a vehicle control group, ^a^Compared to ethanol-induced ulcerated rats, ^b^Compared to Esmo ulcerated group, and ^c^Compared to free Hes ulcerated groups. Each group differed significantly from the others at p ≤ 0.05.

As shown in Figure 10, a significant reduction (83.02%) was observed in anti-inflammatory cytokine activity (IL-10) in ulcerated rats caused by ethanol compared with a vehicle control group. It was found that pre-treatment with Esmo, free Hes, and Nano Hes, significantly increased (120.58%, 135.77%, and 391.17%, respectively) for IL-10 content when compared to ethanol-induced ulcerated rats (Figure 10). Also, Pretreatment with Nano Hes showed a significant increase (122,67%, and 108.33%, respectively) for IL-10 content as compared with Esmo and free Hes ulcerated groups.

### 3.12 Effect of hesperidin (free and encapsulated) on gastric M1 macrophages CD38

**FIGURE 11:**
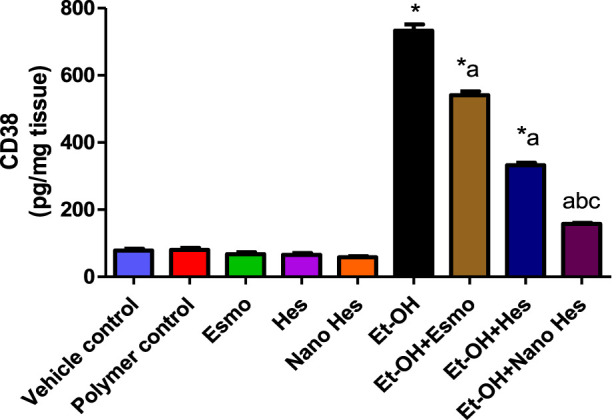
Effect of hesperidin (free and encapsulated) pretreatment on gastric M1 macrophages CD38 content in rats’ ethanol-induced gastric ulcers. Data expressed as mean ± SD (*n* = 6/group). ^*^Compared to a vehicle control group, ^a^Compared to ethanol-induced ulcerated rats, ^b^Compared to Esmo ulcerated group, and ^c^Compared to free Hes ulcerated groups. Each group differed significantly from the others at p ≤ 0.05.

**FIGURE 12:**
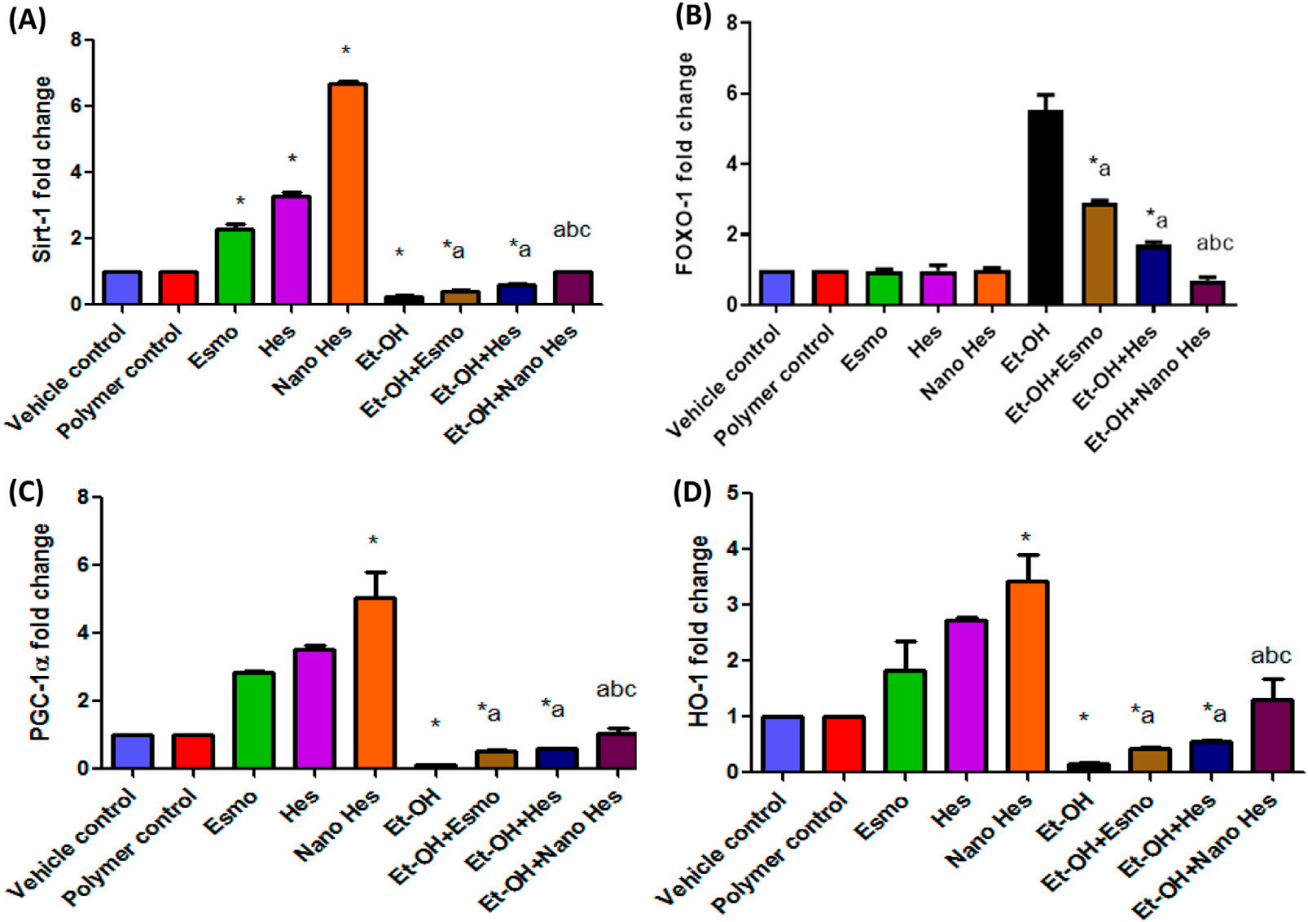
Effect of hesperidin (free and encapsulated) pretreatment on gastric **(A)** mRNA expression of Sirt-1 **(B)** mRNA expression of FOXO-1 **(C)** mRNA expression of PGC-1α **(D)** mRNA expression of HO-1 in ethanol-induced gastric ulcers in rats. Data expressed as mean ± SD (*n* = 6/group). ^*^Compared to a vehicle control group, ^a^Compared to ethanol-induced ulcerated rats group, ^b^Compared to Esmo ulcerated group, and ^c^Compared to free Hes ulcerated groups. Each group differed significantly from the others at p ≤ 0.05.

As shown in Figure 11, a significant elevated (838.46%) was observed in M1 macrophages CD38 content in ulcerated rats caused by ethanol compared with a vehicle control group. It was found that pre-treatment with Esmo, free Hes, and Nano Hes, significantly reduced (26.14%, 54.63%, and 78.53%, respectively) in CD38 content when compared to ethanol-induced ulcerated rats (Figure 12). Also, Pretreatment with Nano Hes showed a significant decrease (70.91%, and 52.69%, respectively) in CD38 content as compared with Esmo and free Hes ulcerated groups.

### 3.13 Effect of hesperidin (free and encapsulated) on gastric mRNA expression of Sirt-1/FOXO-1/PGC-1α/HO-1

As shown in Figure 12, Esmo, Hes, and Nano Hes groups achieved a significant upregulation (130%, 227%, and 570%, respectively) in the mRNA expression level of Sirt-1, a significant upregulation (183.2%, 251%, and 404%, respectively) in gastric levels of the mRNA expression level of PGC-1α and a significant upregulation (183.3%, 172%, and 242.5%, respectively) in mRNA expression level of HO-1 comparing with control group. Oral administration of ethanol experienced a significant downregulation (74%, 86.7%, and 83.6%, respectively) in the mRNA expression level of Sirt-1, PGC-1α, and HO-1, respectively, relative to the vehicle control group. Conversely, pre-treatment with Esmo, free Hes, and Nano Hes, significantly upregulated (63.85%, 130.65%, and 280%, respectively) in the mRNA expression level of Sirt-1, significantly upregulated (303%, 356.39%, and 696.24%, respectively) in the mRNA expression level of PGC-1α and significantly upregulated (161.58%, 234.76%, and 693.9%, respectively) in the mRNA expression level of HO-1 as relative to ethanol-induced ulcerated rats with greater effect in the Nano Hes group. Moreover, Pretreatment with Nano Hes showed significant upregulation (131.92% and 64.94%, respectively) in the mRNA expression level of Sirt-1, significantly upregulated (97.57%, and 74.38%, respectively) of PGC-1α mRNA expression level and significantly upregulated (203.28% and 137.16%, respectively) of HO-1 mRNA expression level as compared with Esmo and free Hes ulcerated groups (Figure 12).

Also, results reported that oral administration of ethanol experienced a significant upregulation in the mRNA expression level of FOXO-1 (452%) relative to the vehicle control. In contrast, Esmo, free Hes, as well as Nano Hes pre-treatments had significantly downregulated (47.64%, 69.16%, and 87.28%, respectively) in the mRNA expression level of FOXO-1 relative to ethanol-induced ulcerated rats with a more significant impact in the Nano Hes group. Moreover, Pretreatment with Nano Hes showed significant downregulation (75.71%, and 58.75%, respectively) for mRNA expression of FOXO-1 compared with Esmo and free Hes ulcerated groups (Figure 12).

### 3.14 Effect of hesperidin (free and encapsulated) on gross morphological changes of rats’ gastric mucosa

**FIGURE 13:**
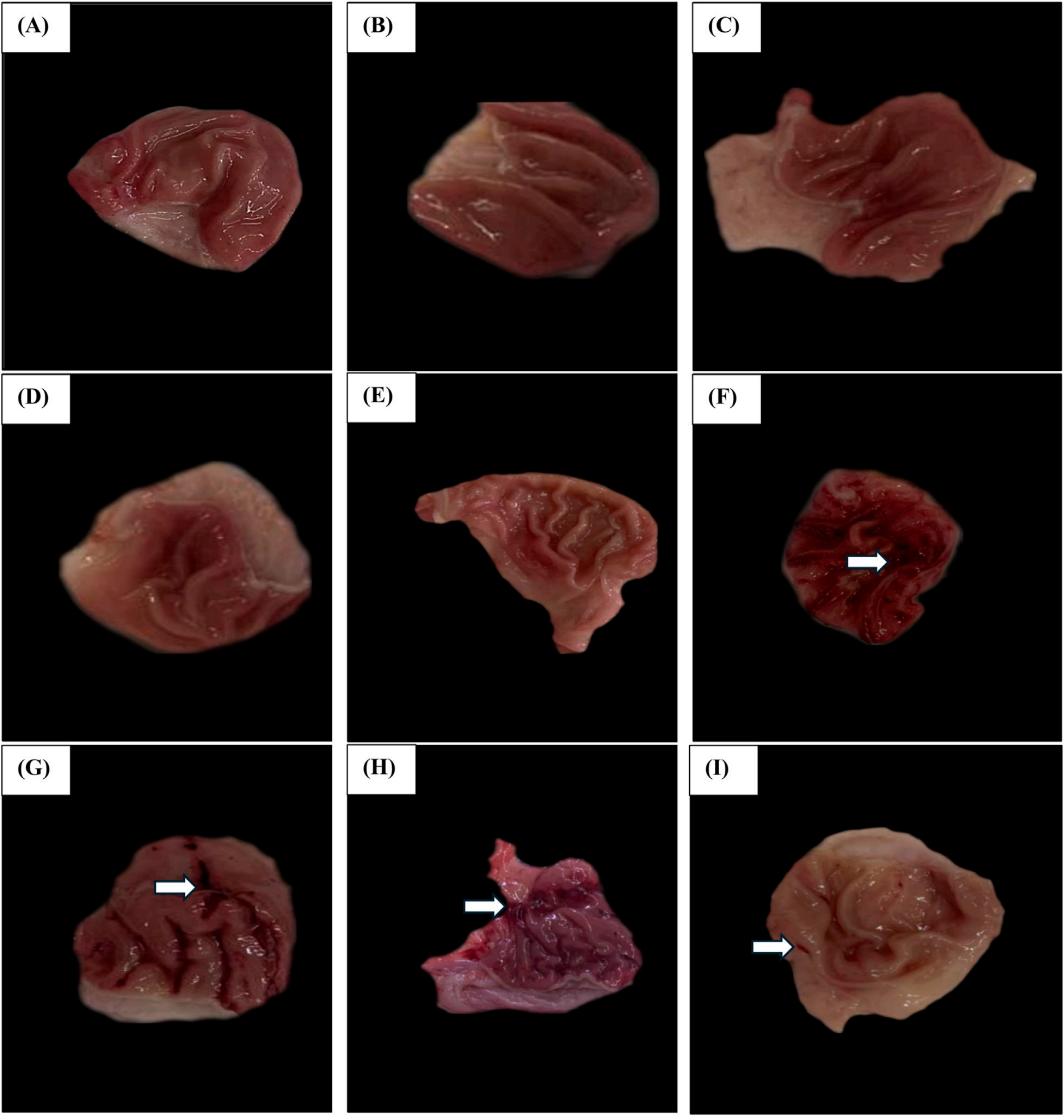
Effect of hesperidin (free and encapsulated) pretreatment Gross Morphological Changes of Rats’ Gastric Mucosa. **(A–E)** Photomicrographs of Vehicle control, Polymer control, Esmo, Hes, and Nano Hes groups show normal gastric mucosa with no significant lesions. **(F)** A photomicrograph of the Ethanol-induced ulcerated group showing extensive and severe hemorrhagic gastric mucosal lesions. **(G,H)** Photomicrographs of Esmo and free Hes pretreated groups showed mild to moderate protection against ethanol-induced gastric damage, with fewer hemorrhagic lesions of the gastric mucosa compared with the control group. **(I)** Photomicrograph of Nano Hes pretreated group showing significant protection with marked recovery as indicated by no hemorrhagic bands or injuries.

Upon morphological examination of the gastric mucosa, Figure 13A illustrates that the Vehicle Control group demonstrated a normal gastric mucosa architecture with an average score of 0.00 ± 0.00, indicating an absence of hemorrhagic lesions or ulceration. Similarly, the Polymer Control (Figure 13B) has an average score of 0.20 ± 0.45, Esmo (Figure 13C) with an average score of 0.20 ± 0.44, Hes (Figure 13D) with an average score of 0.20 ± 0.44, and Nano Hes (Figure 13E) with an average score of 0.00 ± 0.00 groups all presented normal gastric mucosa with minimal or no significant lesions.

In contrast, the Ethanol-induced ulcerated group (Figure 13F) demonstrated severe damage and extensive visible hemorrhagic necrosis, with an average score of 3.60 ± 0.84. The Esmo pretreated group (Figure 13G) exhibited fewer hemorrhagic lesions compared to the ethanol-induced group, with an average score of 2.00 ± 0.71. The Free Hes pretreated group (Figure 13H) showed marked improvement with an average score of 1.20 ± 0.45, indicating fewer lesions. The Nano Hes pretreated group (Figure 13I) demonstrated significant protection, with an average score of 0.40 ± 0.55, reflecting minimal or no visible lesions.

### 3.15 Effect of hesperidin (free and encapsulated) on gastric histopathological changes

As shown in Supplementary Figures S1A–E, Esmo, Hes, and Nano Hes groups demonstrated normal histological characteristics in the stomach tissue, including surface gastric epithelium with normal surface columnar cells. Conversely, the Ethanol-induced ulcerated rats group showed significant sloughing, superficial erosion, and necrosis in the surface gastric epithelium with hemorrhage in the gastric glands, along with parietal cells and chief cells. Moreover, engorged blood vessels in Lamina propia and submucosa (Supplementary Figure S1E). However, It was found that pre-treatment with Esmo, free Hes significantly Protects gastric mucosa with minute superficial erosive necrosis when compared to ethanol-induced ulcerated rats (Supplementary Figures S1G–H). Moreover, Pretreatment with Nano Hes was able to return the histomorphology to normal by stopping additional necrosis with regular gastric glandular, pits, parietal cells, and lamina propria (Supplementary Figure S1I).

### 3.16 Effect of hesperidin (free and encapsulated) on gastric immunohistochemical markers (NF-κB p65, IL-1β, COX-2, Caspase-3, p53, Sirt-1, FOXO1, and CD86)

#### 3.16.1 Inflammatory marker expression

The current investigation investigation delved into the modulation of inflammatory biomarkers following pharmacological intervention in an ethanol-induced gastric ulcer model in rats. Immunohistochemical staining assessed the levels of NF-κB p65, IL-1β, and COX-2, across various treatment groups, including Vehicle Control, Polymer Control, Esmo-treated, Hesperidin-treated, Nanoemulsion Hesperidin-treated, Ethanol-induced ulcerated, and pre-treated groups. Notably, the Ethanol-induced ulcerated group exhibited a pronounced escalation in NF-κB p65 expression, indicative of intense inflammation, which was mitigated by Esmo and Hesperidin pre-treatments, with Nanoemulsion Hesperidin showcasing potent anti-inflammatory effects. Similar trends were observed for IL-1β and COX-2, highlighting the efficacy of pre-treatments in reducing inflammatory responses (Supplementary Figures S2–S4).

#### 3.16.2 Apoptotic and cellular stress marker expression

The apoptotic and cellular stress responses via caspase-3, p53, Sirt-1, and FOXO1 expressions were also elevated. The Ethanol-induced ulcerated group showed heightened caspase-3 and p53 expressions, signifying increased apoptotic activity and cellular stress, respectively, which were significantly reduced by pre-treatment. Nanoemulsion Hesperidin exhibited remarkable cytoprotective effects, normalizing caspase-3 and p53 expressions. Sirt-1 expression dynamics revealed its role in the cellular stress response, with notable increases observed in pre-treated groups. Additionally, upon ethanol challenge, FOXO1 expression increased significantly, suggesting a response to heightened oxidative stress. However, pre-treatments with Esmo and Hesperidin, particularly with Nanoemulsion Hesperidin, effectively reduced FOXO1 levels, indicating robust mitigation of oxidative stress and a protective stabilization of cellular homeostasis (Supplementary Figures S5–S8).

#### 3.16.3 M1 macrophages marker

Immunohistochemical staining assessed the levels of CD86 across various treatment groups, including Vehicle Control, Polymer Control, Esmo-treated, Hesperidin-treated, Nanoemulsion Hesperidin-treated, Ethanol-induced ulcerated, and pre-treated groups. Particularly, the Ethanol-induced ulcerated group displayed a noticeable escalation in CD86 expression, indicative of intense inflammation, which was mitigated by Esmo and Hesperidin pre-treatments, with Nanoemulsion Hesperidin showcasing potent anti-inflammatory effects (Supplementary Figure S9).

## 4 Discussion

A stomach or proximal duodenal ulcer is a digestive ailment that often affects the mucosa lining of these organs (Olatunji et al., 2015). Stress related to stomach ulcers increases the release of catecholamines, cortisol, and gastric acid as well as the creation of inflammatory cytokines that cause tissue damage Karampour et al., 2019).

The experimental model for ethanol-induced stomach ulcers determines the origins of gastric ulcers in humans and, hence, helps identify the anti-ulcer properties of medications in addition to the possible molecular processes at work in this procedure (Karampour et al., 2019; Beiranvand, 2022). Ethanol harms the stomach in numerous ways including dehydration, which compromises mucosal cell defenses, and cytotoxicity (Karampour et al., 2019). This cytotoxicity helps to attract leukocytes that release reactive oxygen species (ROS) and inflammatory mediators, both may cause cell death. Interestingly, NF-κB is important in the connection between these negative outcomes (Vandendriessche et al., 2021).

A citrus bioflavonoid called hesperidin (Hes) mostly found in sweet oranges and lemon has probable anti-inflammatory, antioxidant, anti-cancer, lipid-lowering, neuroprotective, and hypoglycemic properties. According to numerous studies, Hes’s anti-inflammatory actions are mediated by a variation of mechanisms (Guo et al., 2019), such as neutralizing ROS, scavenging free radicals, and boosting cellular antioxidant defenses (Rahmani et al., 2023).

Phytotherapy utilizes bioactive compounds of natural products with fewer incidences of side effects as evidenced by several types of research, showing a beneficial anti-oxidant and anti-inflammatory effect, regulating diabetes, in addition to regulation of cell signaling pathways. Hesperidin is one of the most common and widespread plant phenol chemicals which is recognized for its antioxidant, and anti-inflammatory effects (Rahmani et al., 2023).

Esomeprazole was selected as an over-the-counter model of proton pump inhibitor, used for both treatment and protection against peptic ulcer. Taking esomeprazole for more than a year may increase your chances of certain side effects, including; bone fracture, gut infection, and vitamin B12 deficiency (Hou et al., 2020).

In light of the aforementioned, this study examined the stomach healing effects of hesperidin or its nanoformulation using a gastric ulcer experimental model that most closely reflects the disease in humans.

Natural chemicals’ stability and solubility difficulties can be solved by using natural products conventionally with nanometer-sized objects (Huang et al., 2021). Chitosan polymer has a cytoprotective effect on gastric cells with better ulcer healing (Huang et al., 2021; Sheikh and Bakr, 2023).

Hesperidin nanoparticles were created in the current work using ionotropic gelation (IG), chitosan nanoparticles, sodium tripolyphosphate (STPP), and hesperidin. Chitosan has been used to improve the absorption of advantageous chemical substances like flavonoids (Liang et al., 2017) and provide sustained release action due to the mucoadhesive power of chitosan (Cardoso et al., 2021; Huang et al., 2021). The results revealed that drug release for HNPs follows the Higuchi release model, meaning the release of drug insoluble matrix, which, confirms the sustained release action of HNPs.

The IG method does not require expensive tools or chemicals, and is rapid and reasonably priced (it may take less than 10 h to complete). The synthesis conditions can be altered to produce nanoparticles or microparticles by changing the ratio of the reagents, adding a stabilizer, or using other techniques. The use of water-based solutions is another factor to take into account while looking for bio-safe procedures (Wu et al., 2023). Beyond the bioavailability of drug formulations, which is greatly increased by encapsulation, the IG method has advantages. A high encapsulation efficiency of nearly 100% is reached when interactions between the polymer and the medicine are perfect (Negi and Kesari, 2022).

It is believed that particles of a size of 200–300 nm are adequate for navigating biological barriers and evading glomerular filtration (Umair et al., 2016). Particle sizes of the produced HNPs in this work were in the nanosize range (128.7 nm–487.5 nm). The polydispersity index (PDI) of the created nanoparticles was always less than 0.5. The HNPs were PDI = 0.408 ± 0.020. The size of the system affects the interaction with tissues or particular cell structures and affects pharmacokinetics and clearance in drug delivery systems using nanoparticles (Danaei et al., 2018; Mahmood et al., 2019). Otherwise, FTIR spectra of HNPs had a small change that corresponded to a peak at 3,506 with greater intensity; this was likely caused by the formation of hydrogen bonds with chitosan (Ali et al., 2019). The superior solubility of HNPs can be explained by the nano-sized and nano-sphere which enhance solubility by increasing the exposed surface according to the New Whitney equation. The ability to form hydrogen bonds in addition to the swelling character of chitosan may be responsible for the enhanced solubility of Hesperidin (Ali et al., 2019; Mahmood et al., 2019).

Consumption of ethanol specifically produces a variety of pathological alterations to the stomach like hemorrhagic lesions, substantial submucosal edema, and mucosal friability. Additionally, ethanol can cause local ischemia of the stomach mucosa, direct disturbance of cell stability, generation of reactive oxygen radicals and inflammatory cytokines, and more (Kim et al., 2018; Liu et al., 2018; Simões et al., 2019).

Based on this study, ulcers caused by ethanol showed higher stomach acidity and decreased gastric pH. Moreover, all oxidative stress indicators, such as MDA and NO, as well as mediators of inflammatory processes like NF-κB p65, TNF-α, HO-1, MPO, and IL-1β and apoptotic markers caspase-3 and P53 were also clearly elevated. On the other hand, antioxidant indicators SOD and CAT in addition to anti-inflammatory IL-10 were measured. Furthermore, M1 macrophage markers CD86 and CD38 were also determined.

These outcomes had been confirmed by histopathological analysis that revealed sloughing, superficial erosion, and necrosis in the surface gastric epithelium with hemorrhage in the gastric glands along with parietal cells with engorged blood vessels in lamina propia and submucosa.

The transcription regulator (NF-κB subfamily) plays an important role in controlling the inflammatory response and oxidative stress which is in charge of interaction with restrictive proteins and DNA binding (Linggapan, 2018; Hopkins and Neumann, 2019).

A crucial step in the stomach mucosa defense system is the inflammatory response (Jia et al., 2023). Ethanol promotes inflammation sets off the activation of macrophages and accelerates the generation of inflammatory cytokines. These results were consistent with an immunohistochemical finding of a strong positive immune reaction of NF-κB p65 and IL-1β and decreased anti-inflammatory cytokines like IL-10 which encourages the accretion of neutrophils, breakdown of mucosal barriers along with the creation of ROS (Almasaudi et al., 2016; Badr et al., 2019). Moreover, ethanol intubation elevated the level of MPO, a sign of neutrophil infiltration (Badr et al., 2019).

Oxidative stress can be caused by a redox imbalance between the production of ROS and their scavenging by antioxidant defenses (Jomova et al., 2024). In the meantime, neutrophils (O2•−) produced in the ulcerated stomach tissues interact with lipids to cause lipid peroxidation (Andrés Juan et al., 2021). Lack of the redox balance that is heightened during lipid peroxidation was the cause of the loss of antioxidant enzymes including GSH, SOD, and CAT.

The health of the stomach’s mucosal lining wall is significantly disrupted by the ROS-induced apoptosis regulated through pro-apoptotic Bax and caspase-3 proteins (Chen, 2016; Liu et al., 2018). Many earlier studies demonstrated that oxidative stress and inflammatory cytokines cause stomach mucosal apoptotic damage (Fiorucci et al., 1998; Martel-Pelletier et al., 2003; Ghanim et al., 2010).

Likewise, the activation of NF-κB in the stomach as a response to ROS increases TNF-α gastric content which leads to raised expression of both COX-2 and iNOS and leukocyte adhesion boosted by increased COX-2 production (Jomova et al., 2023; Piacenza et al., 2022). Stomach ulceration results from COX-2 increased neutrophil activation and decreased stomach mucosal epithelial cell regeneration (Liu et al., 2021). Whereas elevated iNOS expression causes NO to be produced, which then combines with superoxide to produce peroxynitrites (Jomova et al., 2023; Piacenza et al., 2022) and significant harm to the gastrointestinal mucosa, as demonstrated by the most recent research.

This study noted that pretreatment with Esmo, free Hes, and HNPs, significantly inhibited total acidity and increased gastric pH confirmed by improving mucosal histopathological erosions, necrosis, and hemorrhage. Besides, all oxidative stress indicators, like MDA and NO, as well as inflammatory mediators like NF-κB p65, TNF-α, and IL-1β as well as MPO enzyme activity, and apoptotic markers caspase-3 and P53 were also clearly suppressed. On the other hand, antioxidant indicators SOD and CAT in addition to anti-inflammatory IL-10 were increased. Furthermore, pretreatment with Nano Hes provides the greatest protection than free Hes and Esmo. These results were consistent with restoring histopathological damage. In addition, a negative immune stain of inflammatory markers and apoptotic markers. These outcomes are in line with those of earlier reports. Additionally, previous research has shown that elevated levels of antioxidants prevent NF-κB initiation driven by ROS and impede the production of numerous cytokines that are (Kozlov et al., 2024).

In the same context, the current study found that the M1 macrophage markers CD38 and CD86 increased as a response to ethanol-induced ulcers. Instead, Pretreatment with Esmo, free Hes, and principally Nano Hes caused a reduction in the CD38 and CD86 content.

Macrophages are a diverse group of mononuclear phagocytes that have undergone differentiation (Dash et al., 2024). They are engaged in every stage of the immune response, from the beginning of the response and the induction of adaptive immunity to the resolution of inflammation and tissue repair. Moreover, macrophages are critical for tissue remodeling, development, and homeostasis (Chen et al., 2023).

The Th1/Th2 paradigm, which was already widely accepted, was linked to the original concept of M1 and M2 macrophages, which were based on distinct metabolic programs and related to previously described “classically” and “alternatively” activated macrophages. These concepts were widely used in both experimental and clinical settings (Viola et al., 2019).

High concentrations of pro-inflammatory cytokines, including IL-1β, IL-6, TNF-α, and IFNγ, can be released by M1 macrophages (Gajanayaka et al., 2021) and they can also secrete IL-12 to trigger Th1 differentiation (Zhou et al., 2021). The activation of M1 macrophages and pro-inflammatory cytokines caused inhibition in anti-inflammatory cytokines, such as IL-10 and TGF-β which participate in the remodeling and repair of tissue and are crucial in the inhibition of the immunological response (Jiang et al., 2021).

Likewise, this study found that gene expression of Sirt-1, PGC-1α, and HO-1 had been downregulated while FOXO1 expression was upregulated as a response to ethanol-induced ulcers. On the other hand, Pretreatment with Esmo, free Hes, and particularly Nano Hes caused upregulated expression of Sirt-1, PGC-1α, and HO-1 along with downregulated FOXO1 expression. These outcomes support earlier discoveries (Tahir et al., 2017; Panes et al., 2020).

Sirt1 might inhibit NF-κB activation the main chief regulator of the production of pro-inflammatory cytokines, and control P53 and caspase-3 signaling which would have a crucial role in the control of oxidative stress, inflammation, and apoptosis (Jiang et al., 2021). Besides, through phosphorylation and deacetylation, SIRT1 may directly influence the PGC-1α function (Qian et al., 2024). PGC-1α and other PGC-1 family members are powerful stimulators of mitochondrial respiration and gene transcription that can reduce oxidative stress, inflammation, and apoptosis (Panes et al., 2020). In the same manner, HO-1 is one of the most strongly associated with the protective impact on the gastrointestinal system which may either directly offer cytoprotection against oxidative stress and protect against apoptosis or do so by suppressing ROS (Puentes-Pardo et al., 2020). Alongside, one of the FOXO family’s isoforms, FOXO1, can stimulate the activation of NF-κB downstream cytokines such as IL-6 and TNF-α. When FOXO1 expression is downregulated, NF-κB releasing inflammatory mediators is inhibited as a result. Moreover, it has been noted that Sirt-1 regulates the inflammatory response by targeting FOXO1/NF-κB (Graves and Milovanova, 2019; Kim et al., 2022; Wu et al., 2022).

To the best of our knowledge, no studies investigated the protective properties of HNPs against rats’ ethanol-induced gastric ulcers in an animal model. These results suggest the potential beneficial effects of HNPs in mitigating gastric ulcers.

## 5 Conclusion

The gastroprotective properties of hesperidin against ethanol-induced stomach ulcers are greatly enhanced by chitosan nanoparticles, which may provide a more successful treatment approach. HNPs significantly increased the activity of antioxidant enzymes, decreased oxidative stress and inflammatory markers, and more successfully improved histology findings. As far as we are aware, this work is the first to shed light on the mechanism of action and potential protective impact of HNPs in the stomach ulcer model, offering efficient natural product-based therapies for stomach ulcers could open up new possibilities. The therapeutic uses of HNPs in human patients should be investigated further in future studies.

## Data Availability

The original contributions presented in the study are included in the article/Supplementary Material, further inquiries can be directed to the corresponding author.
