# Supplementary material for: Chitosan/hesperidin nanoparticles formulation: a promising approach against ethanol-induced gastric ulcers via Sirt1/FOXO1/PGC-1α/HO-1 pathway
Source: Front Pharmacol. 2024 Sep 9;15:1433793. doi: 10.3389/fphar.2024.1433793 (PMC11417028; doi:10.3389/fphar.2024.1433793)
Supplement: Supplementary file 1 [file DataSheet1.PDF]

Supplementary Material

Chitosan/hesperidin nanoparticles protect against Ethanol-Induced Gastric Ulcers: The Innovative Anti-Ulcer Strategies that Target The route of Sirt-1/FOXO1/PGC-1α/HO-1 signaling

Jawaher Abdullah Alamoudi[1], Thanaa A. El-Masry[2], Maysa M. F. El-Nagar[2,*], Enas I. El Zahaby[3], Kadreya E. Elmorshedy [4], Mohamed M. S. Gaballa[5], Samar Zuhair Alshawwa[1], Maha Alsunbul[1], Sitah Alharthi[6], Hanaa A. Ibrahim[2]

[1]Department of Pharmaceutical Sciences, College of Pharmacy, Princess Nourah bint Abdulrahman University, P.O.Box 84428, Riyadh 11671, Saudi Arabia; Jaalamoudi@pnu.edu.sa **(J.A.A.-A),** szalshawwa@pnu.edu.sa **(S.Z.A);** Maalsonbel@pnu.edu.sa **(M.A.-S)**

[2]Department of Pharmacology and Toxicology, Faculty of Pharmacy, Tanta University, Tanta 31527, Egypt; thanaa.elmasri@pharm.tanta.edu.eg **(T.A.E.-M);** maysa_elnagar@outlook.com **(M.M.F.E.-N);** hanaa.abdelkareem@pharm.tanta.edu.eg **(H.A.I)**

[3]Department of Pharmaceutics, Faculty of Pharmacy, Delta University for Science and Technology, Gamasa 35712, Egypt
ElZaha_bi@deltauniv.edu.eg **(E.I.E.Z.)**

[4]Department of Anatomy, Faculty of Medicine, Tanta University, Tanta 31527, Egypt; Department of Anatomy, King Khaled College of Medicine, Saudia Arabia; Kadreyaelmorshedy@med.tanta.edu.eg **(K.E.E-M)**

[5]Department of Pathology, Faculty of Veterinary Medicine, Benha University, Toukh 13736, Egypt; Mohamed.gaballah@fvtm.bu.edu.eg **(M.M.S.G)**

[6]Pharmaceutical Science Department, College of Pharmacy, Shaqra University, Shaqra 11961, Saudi Arabia; S_alharthi@su.edu.sa **(S.A)**

***Corresponding Authors:** maysa_elnagar@outlook.com

Orchid ID
Jawaher Abdullah Alamoudi, (0000-0003-1776-9249) (orcid.org)
Thanaa A. El-Masry, (0000-0002-1453-5597) (orcid.org)
Maysa M. F. El-Nagar, (0000-0001-5807-1117) (orcid.org)
Enas I. El Zahaby, (0000-0002-1322-8309) (orcid.org)
Kadreya E. Elmorshedy, (0000-0003-2983-7560) (orcid.org)
Mohamed M. S. Gaballa' (0000-0003-2949-430X) (orcid.org)
Samar Zuhair Alshawwa, (0000-0001-9232-7956) (orcid.org)
Maha Alsunbul, (0000-0002-4708-4147) (orcid.org)
Sitah Alharthi (0000-0003-0031-5654) (orcid.org)
Hanaa A. Ibrahim, (0000-0002-9278-314X) (orcid.org)

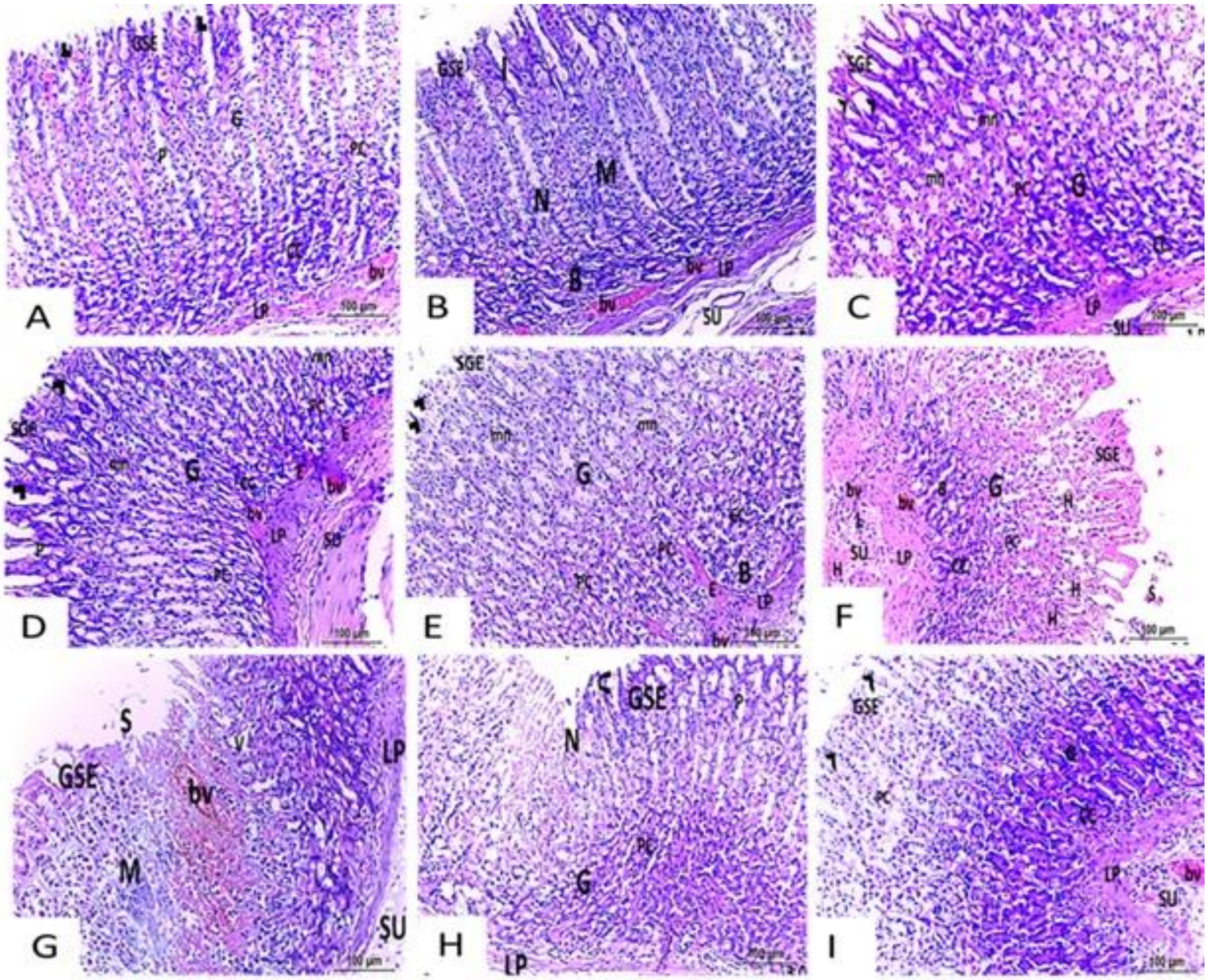
A
B
C
D
E
F
G
H
I

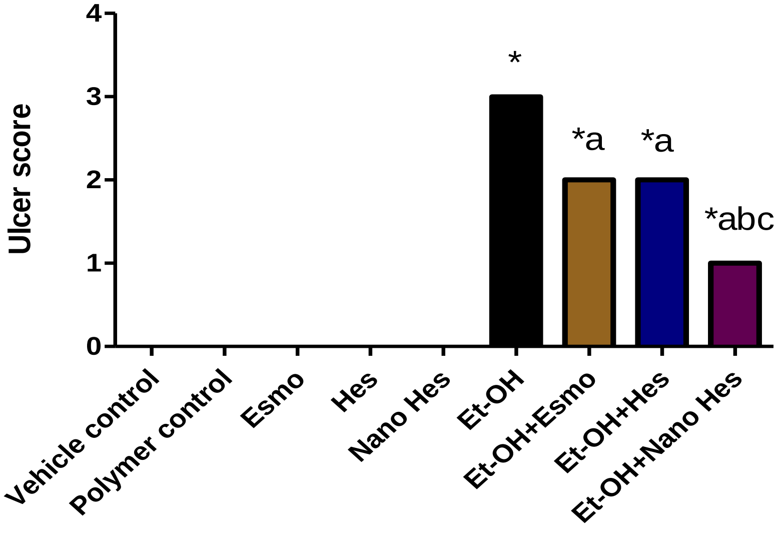
Ulcer score
0
1
2
3
4
*
*a
*a
*abc
Vehicle control
Polymer control
Esmo
Hes
Nano Hes
Et-OH
Et-OH+Esmo
Et-OH+Hes
Et-OH+Nano Hes

Figure 1S. Effect of hesperidin (free and encapsulated) on gastric histopathological changes (200x, Scale bar = 100μm). (A-E) photomicrographs of Vehicle control, Polymer control, Esmo, Hes, and Nano Hes groups showing normal mucosa made up of packed glands made of isthmus, neck, and base. The columnar epithelium with basal nuclei that lines the healthy surface gastric epithelium is seen. The submucosa and lamina propria both have visible blood vessels. (F) A photomicrograph of the Ethanol-induced ulcerated group showing fundic gastric mucosa with sloughing, superficial erosion, and necrosis in the surface gastric epithelium. Large-scale hemorrhage in the gastric glands is evident, along with parietal cells and chief cells. Lamina propia and submucosa are two tissues that exhibit engorged blood vessels. (G-H) photomicrographs of Esmo and free Hes pretreated groups showing minute superficial sloughed necrosis, and congested mucosal blood vessels. It was possible to find lamina propria, normal submucosa, parietal cells, and typical gastric pits. (I) photomicrograph of Nano Hes pretreated group showing normal columnar epithelium (arrowheads) lining the gastric surface epithelium. In addition, mucosal glands parietal cells, and chief cells are seen. There are visible lamina propria and submucosal blood vessels. (M) mucosa, (G) glands, (I) isthmus, (N) neck, (B) base, (SGE) surface gastric epithelium, (SU) submucosa, (LP) lamina propria, (S) sloughing, (H) hemorrhage, (PC) parietal cells, (CC) chief cells, sloughed necrosis (SN), (bv) blood vessels.

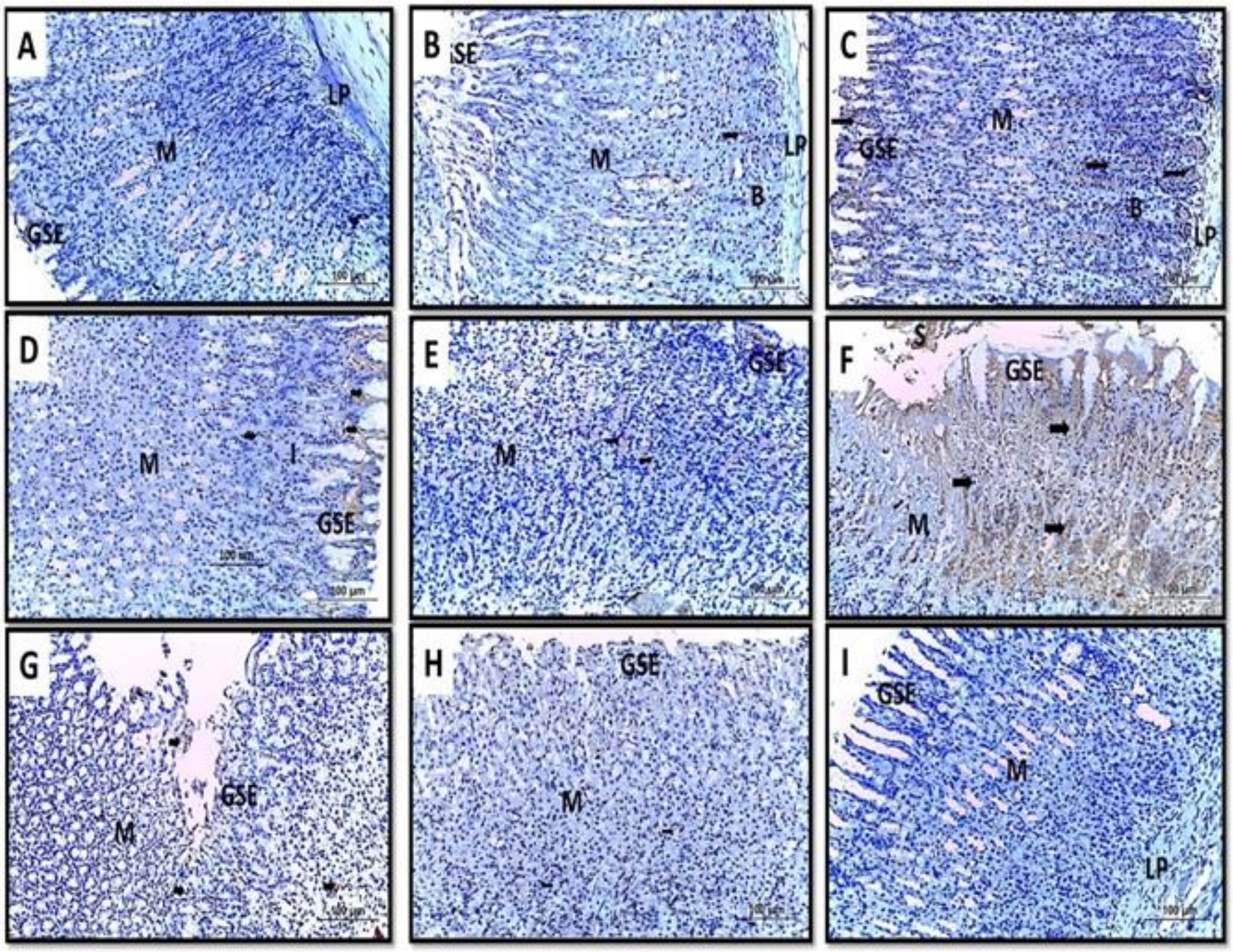
A
LP
M
GSE
B
SE
M
LP
B
C
M
GSE
B
LP
D
M
GSE
E
GSE
M
F
S
GSE
M
G
M
GSE
H
GSE
M
I
GSE
M
LP

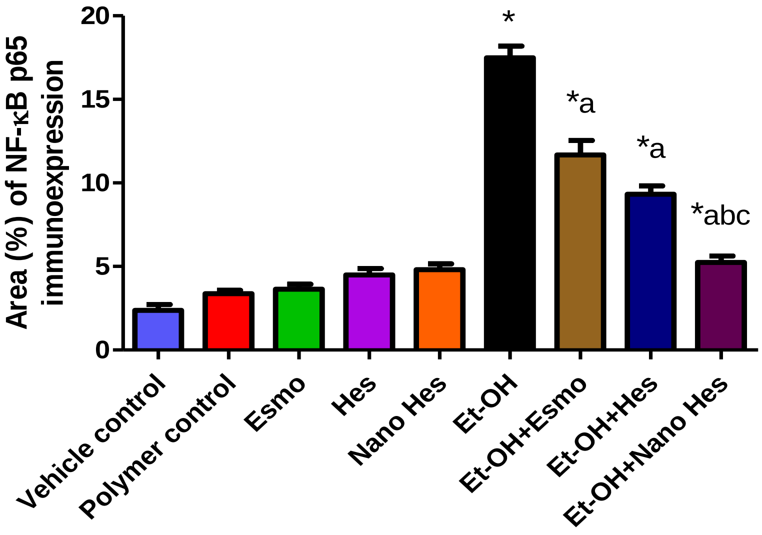
Area (%) of NF-κB p65 immunoexpression
20
15
10
5
0
*
*a
*a
*abc
Vehicle control
Polymer control
Esmo
Hes
Nano Hes
Et-OH
Et-OH+Esmo
Et-OH+Hes
Et-OH+Nano Hes

Figure 2S. NF-κB p65 immunohistochemical analysis of rats' stomach (200x, Scale bar = 100μm). Control groups (Vehicle and Polymer) along with those receiving Esmo, Hes, and Nanoemulsion Hesperidin treatments exhibited minimal NF-κB p65 immunoreactivity, suggesting subdued inflammatory activity under non-pathological conditions (Figures A-E). In stark contrast, ethanol-induced ulceration resulted in a significant increase in NF-κB p65 expression, indicative of an acute inflammatory response (Figure F). Notably, Esmo and Hesperidin pre-treatments attenuated this inflammatory marker to a milder elevation (Figures G-H), while Nanoemulsion Hesperidin pre-treatment aligned NF-κB p65 expression closely with control levels (Figure I), highlighting its potent anti-inflammatory effect. Data expressed as mean ± SD (*n*= 6/group). [*] Compared to a vehicle control group, [a] Compared to ethanol-induced ulcerated rats group, [b] Compared to Esmo ulcerated group, and [c] Compared to free Hes ulcerated groups. Each group differed significantly from the others at $p \leq 0.05$. (M) mucosa, (G) glands, (I) isthmus, (N) neck, (B) base, (SGE) surface gastric epithelium, (SU) submucosa, (LP) lamina propria, (S) sloughing, (H) hemorrhage, (PC) parietal cells, (CC) chief cells, sloughed necrosis (SN), (bv) blood vessels.

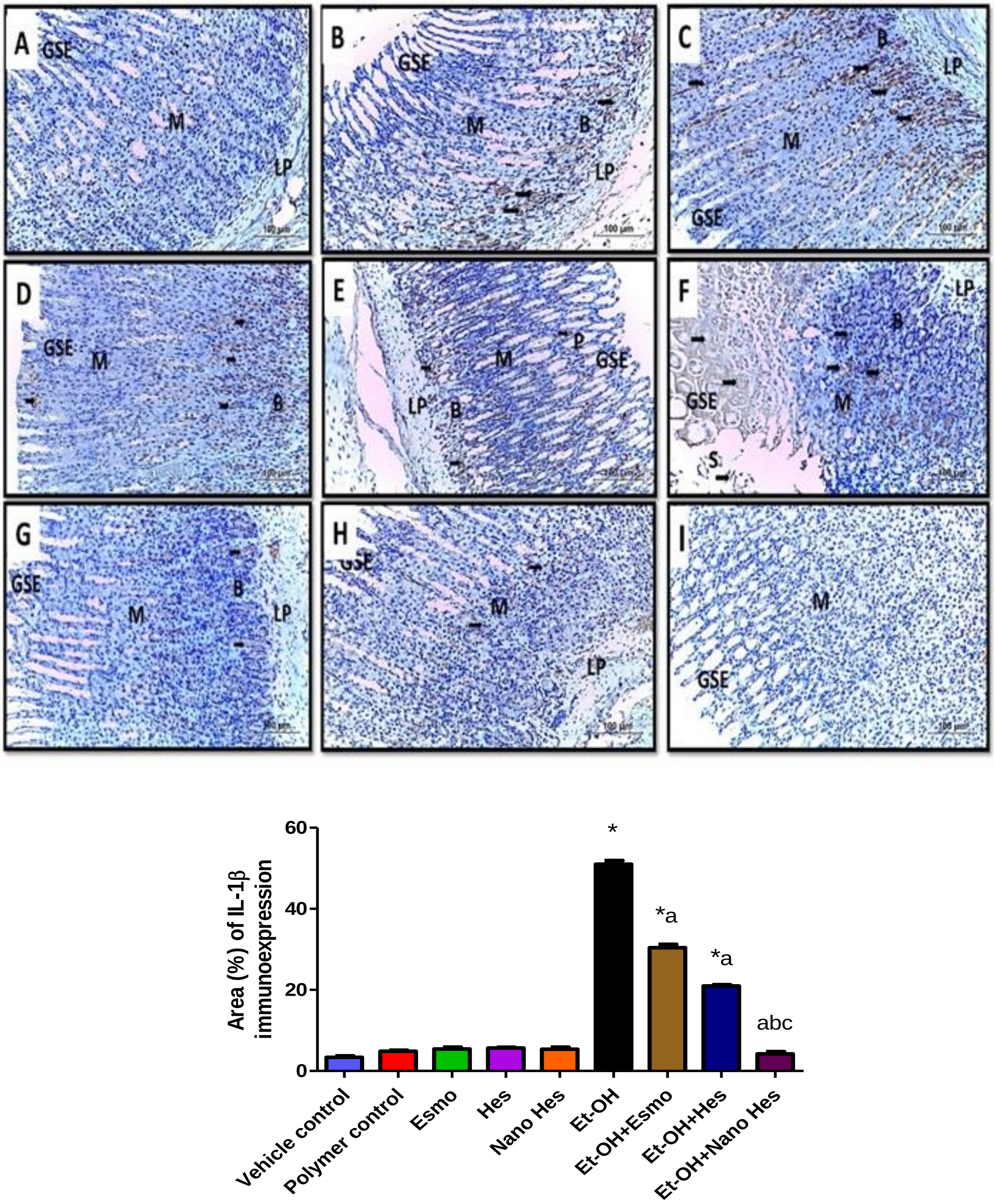

Figure 3S. IL-1β immunohistochemical analysis of rats' stomachs (200x, Scale bar = 100μm). Minimal IL-1β immunoreactivity was observed in the control and Esmo, Hes, and Nanoemulsion Hesperidin-treated

cohorts (Figures A-E). The ethanol challenge precipitated a pronounced elevation in IL-1β expression (Figure F), markedly diminished by Esmo and Hesperidin pre-treatments (Figures G&H). Remarkably, Nanoemulsion Hesperidin pre-treatment demonstrated an efficacious reduction in IL-1β, returning levels to near-baseline (Figure I). Data expressed as mean ± SD (*n*= 6/group). [*] Compared to a vehicle control group, [a] Compared to ethanol-induced ulcerated rats group, [b] Compared to Esmo ulcerated group, and [c] Compared to free Hes ulcerated groups. Each group differed significantly from the others at $p \leq 0.05$. (M) mucosa, (G) glands, (I) isthmus, (N) neck, (B) base, (SGE) surface gastric epithelium, (SU) submucosa, (LP) lamina propria, (S) sloughing, (H) hemorrhage, (PC) parietal cells, (CC) chief cells, sloughed necrosis (SN), (bv) blood vessels.

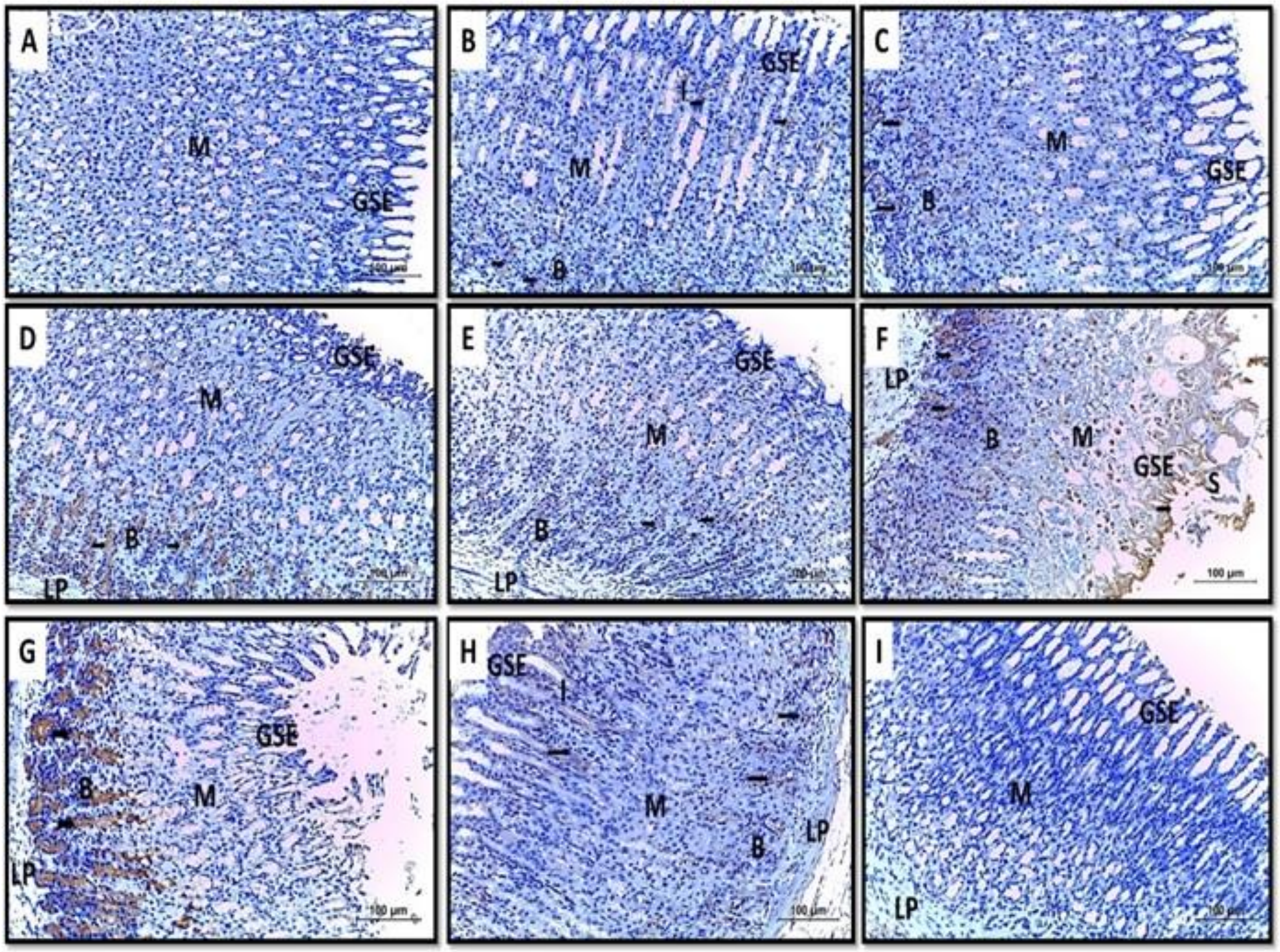

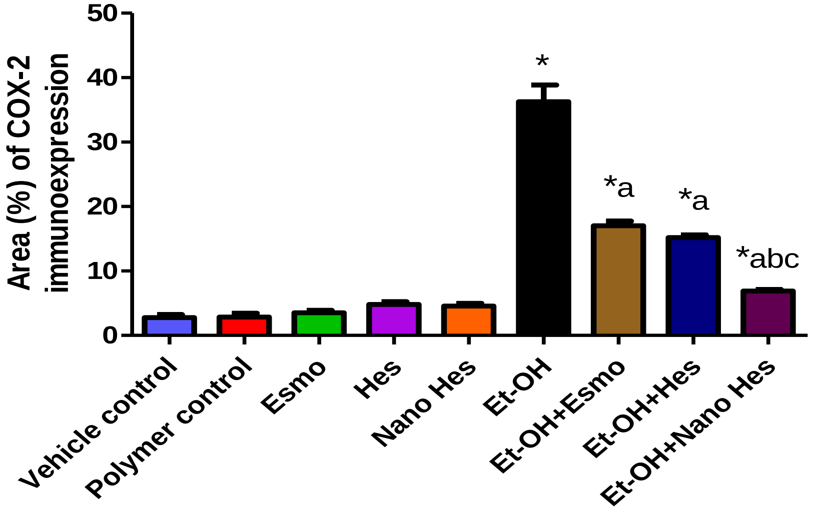

Figure S4. Cycloxygenase-2 (COX-2) immunohistochemical analysis of rats' stomach (200x, Scale bar = 100μm). The Vehicle Control and Polymer Control, along with the Esmo, Hes, and Nano Hes treatments,

showed negligible immunoexpression of COX-2 (Figures A-E), congruent with an intact gastric mucosa. Contrastingly, ethanol exposure significantly escalated COX-2 expression (Figure F), with pre-treatments of Esmo and Hesperidin markedly mitigating this effect (Figures G&H). Nano Hesperidin pre-treatment effectuated a notable diminution of COX-2 expression, suggesting considerable anti-inflammatory properties (Figure I). Data expressed as mean ± SD (*n*= 6/group). [*] Compared to a vehicle control group, [a] Compared to ethanol-induced ulcerated rats group, [b] Compared to Esmo ulcerated group, and [c] Compared to free Hes ulcerated groups. Each group differed significantly from the others at $p \leq 0.05$. (M) mucosa, (G) glands, (I) isthmus, (N) neck, (B) base, (SGE) surface gastric epithelium, (SU) submucosa, (LP) lamina propria, (S) sloughing, (H) hemorrhage, (PC) parietal cells, (CC) chief cells, sloughed necrosis (SN), (bv) blood vessels.

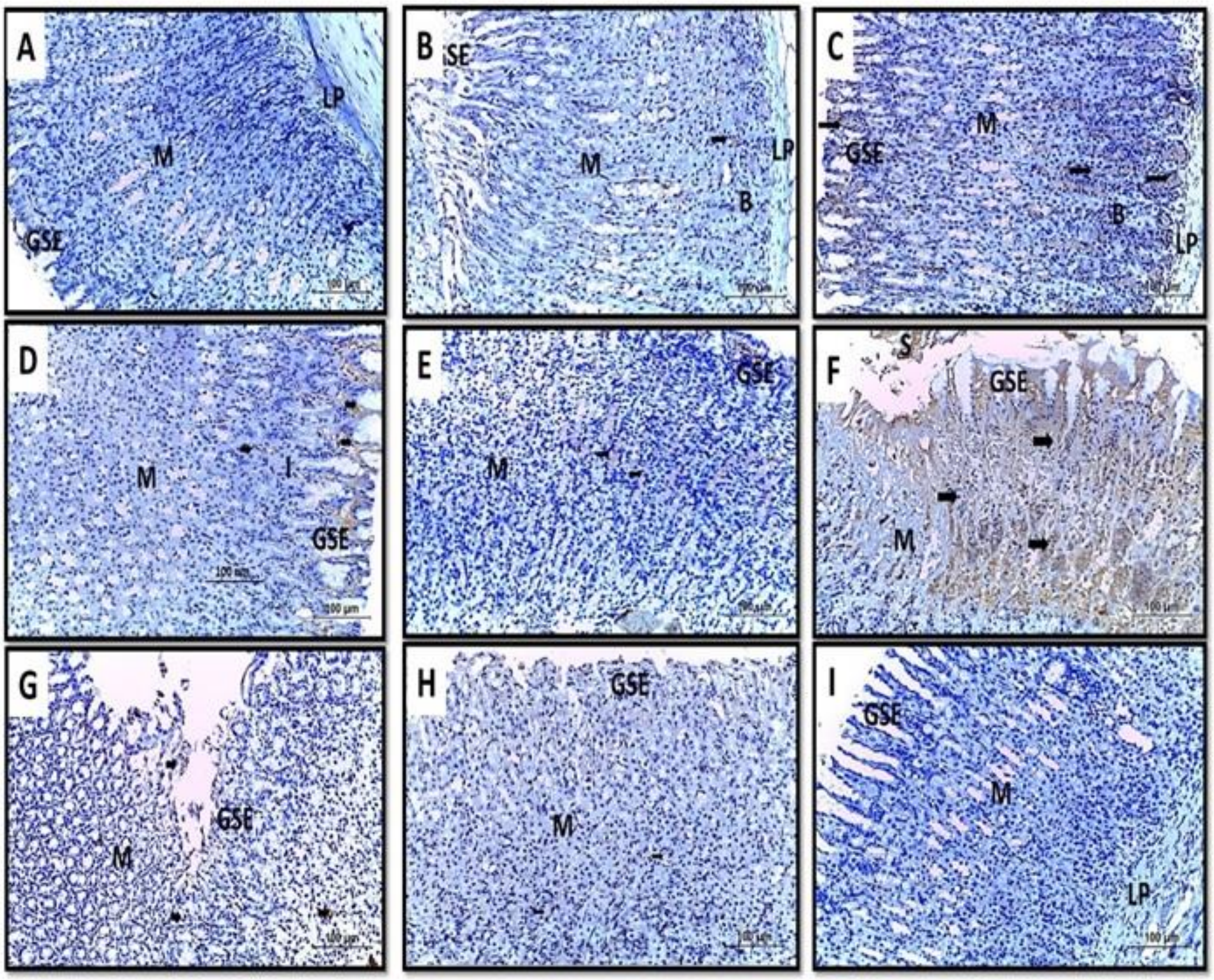
A
LP
M
GSE
B
SE
M
LP
B
C
M
GSE
B
LP
D
M
I
GSE
E
GSE
M
F
S
GSE
M
G
M
GSE
H
GSE
M
I
GSE
M
LP

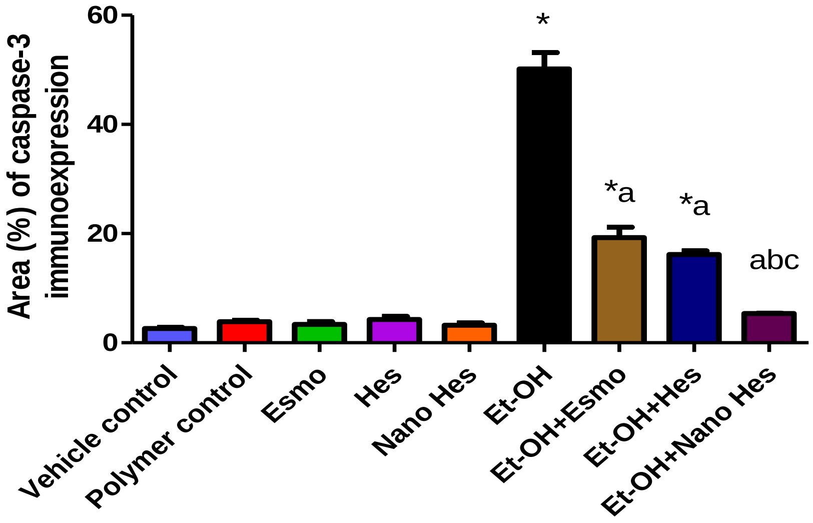
Area (%) of caspase-3 immunoexpression
60
40
20
0
*
*a
*a
abc
Vehicle control
Polymer control
Esmo
Hes
Nano Hes
Et-OH
Et-OH+Esmo
Et-OH+Hes
Et-OH+Nano Hes

Figure S5. Caspase-3 immunohistochemical analysis of rats' stomach (200x, Scale bar = 100μm). In the context of the apoptotic activity, as indexed by caspase-3 expression, control conditions maintained minimal expression levels (Figures A-E). However, the Ethanol-induced ulcerated group displayed a substantial upsurge in caspase-3 (Figure F), substantially reduced following Esmo and Hesperidin pre-treatments (Figure G&H). The Nano Hesperidin pre-treated group displayed a return to baseline caspase-3 expression (Fig. I), suggesting a significant cytoprotective effect. Data expressed as mean ± SD (*n*= 6/group). [*] Compared to a vehicle control group, [a] Compared to ethanol-induced ulcerated rats group, [b] Compared to Esmo ulcerated group, and [c] Compared to free Hes ulcerated groups. Each group differed significantly from the others at p ≤ 0.05. (M) mucosa, (G) glands, (I) isthmus, (N) neck, (B) base, (SGE) surface gastric epithelium, (SU) submucosa, (LP) lamina propria, (S) sloughing, (H) hemorrhage, (PC) parietal cells, (CC) chief cells, sloughed necrosis (SN), (bv) blood vessels.

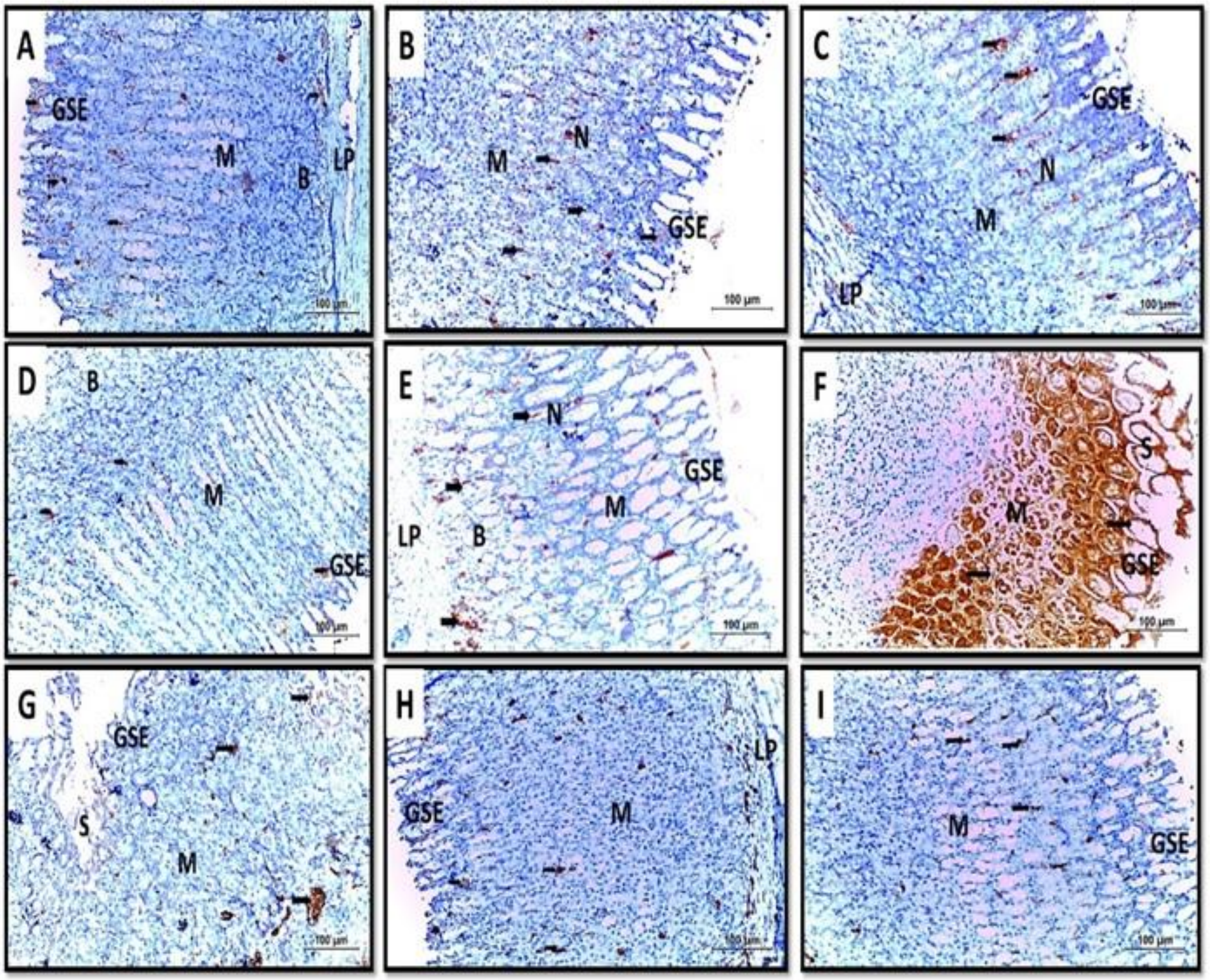
A
GSE
M
B
LP
100 μm
B
N
M
GSE
100 μm
C
GSE
N
M
LP
D
B
M
GSE
E
N
GSE
M
LP
B
100 μm
F
S
M
GSE
100 μm
G
GSE
S
M
100 μm
H
LP
GSE
M
I
M
GSE
100 μm

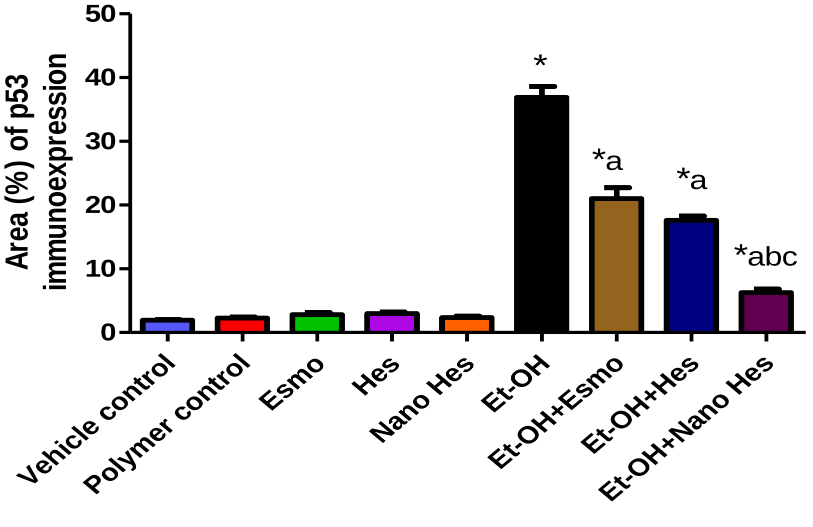
Area (%) of p53 immunoexpression
50
40
30
20
10
0
*
*a
*a
*abc
Vehicle control
Polymer control
Esmo
Hes
Nano Hes
Et-OH
Et-OH+Esmo
Et-OH+Hes
Et-OH+Nano Hes

Figure S6. P53 immunohistochemical analysis of rats' stomachs (200x, Scale bar = 100µm) p53 levels were assayed as an indicator of cellular stress and apoptotic signaling. Comparable to other biomarkers, minimal p53 expression was noted in the control and treated groups (Figures A-E). The ethanol-induced group displayed a significant rise in p53 immunoreactivity (Figure F), indicative of cellular stress. Pre-treatment with Esmo (Figure G) and Hesperidin (Figure H) decreased p53 expression, while Nano Hesperidin pre-treatment notably normalized p53 levels (Figure I), suggesting modulation of stress and apoptotic pathways. Data expressed as mean ± SD (*n*= 6/group). [*] Compared to a vehicle control group, [a] Compared to ethanol-induced ulcerated rats group, [b] Compared to Esmo ulcerated group, and [c] Compared to free Hes ulcerated groups. Each group differed significantly from the others at $p \leq 0.05$. (M) mucosa, (G) glands, (I) isthmus, (N) neck, (B) base, (SGE) surface gastric epithelium, (SU) submucosa, (LP) lamina propria, (S) sloughing, (H) hemorrhage, (PC) parietal cells, (CC) chief cells, sloughed necrosis (SN), (bv) blood vessels.

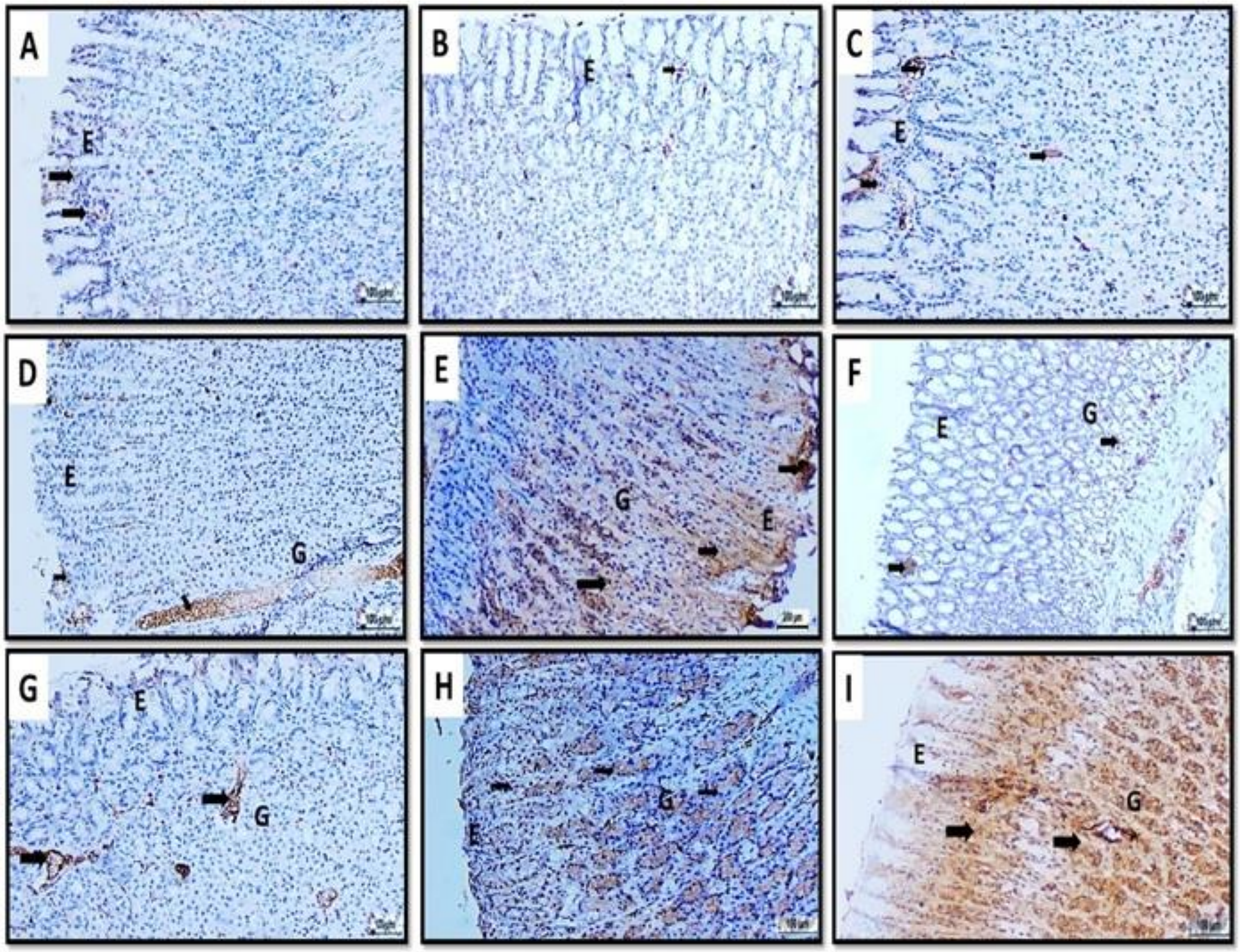
A
E
B
E
C
E
D
E
G
E
G
E
F
E
G
G
E
G
H
G
E
I
E
G

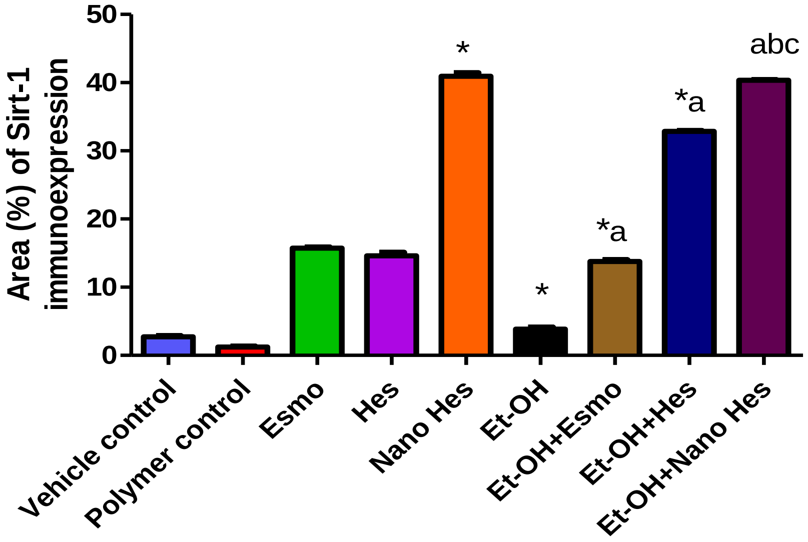
Area (%) of Sirt-1 immunoexpression
50
40
30
20
10
0
*
abc
*a
*
*a
Vehicle control
Polymer control
Esmo
Hes
Nano Hes
Et-OH
Et-OH+Esmo
Et-OH+Hes
Et-OH+Nano Hes

Figure S7. Sirt-1 immunohistochemical analysis of rats' stomachs (200x, Scale bar = 100μm Baseline Sirt1 expression levels in the Vehicle and Polymer Control groups served as reference points for subsequent analyses (Figures A-B). Treatment with Esmo (Figure C) and Hesperidin (Figure D) induced minor elevations in Sirt-1 expression, maintaining consistency with control values. In contrast, the Nano Hesperidin group (Figure E) exhibited a notable increase in Sirt-1 expression, surpassing control levels significantly. Unexpectedly, the Ethanol-induced ulcerated group (Figure F) displayed a decrease in Sirt-1 expression, indicative of downregulation in response to ethanol-induced stress. Esmo pre-treatment (Figure G) significantly enhanced Sirt-1 expression compared to the ethanol-only group. Similarly, Hesperidin pre-treatment (Figure H) led to a more pronounced increase in expression. Notably, the Nano Hesperidin pre-treated group (Figure I) exhibited even higher levels of Sirt-1 expression compared to the ethanol-only condition. Data expressed as mean ± SD (*n*= 6/group). [*] Compared to a vehicle control group, [a] Compared to ethanol-induced ulcerated rats group, [b] Compared to Esmo ulcerated group, and [c] Compared to free Hes ulcerated groups. Each group differed significantly from the others at $p \leq 0.05$. (M) mucosa, (G) glands, (I) isthmus, (N) neck, (B) base, (SGE) surface gastric epithelium, (SU) submucosa, (LP) lamina propria, (S) sloughing, (H) hemorrhage, (PC) parietal cells, (CC) chief cells, sloughed necrosis (SN), (bv) blood vessels.

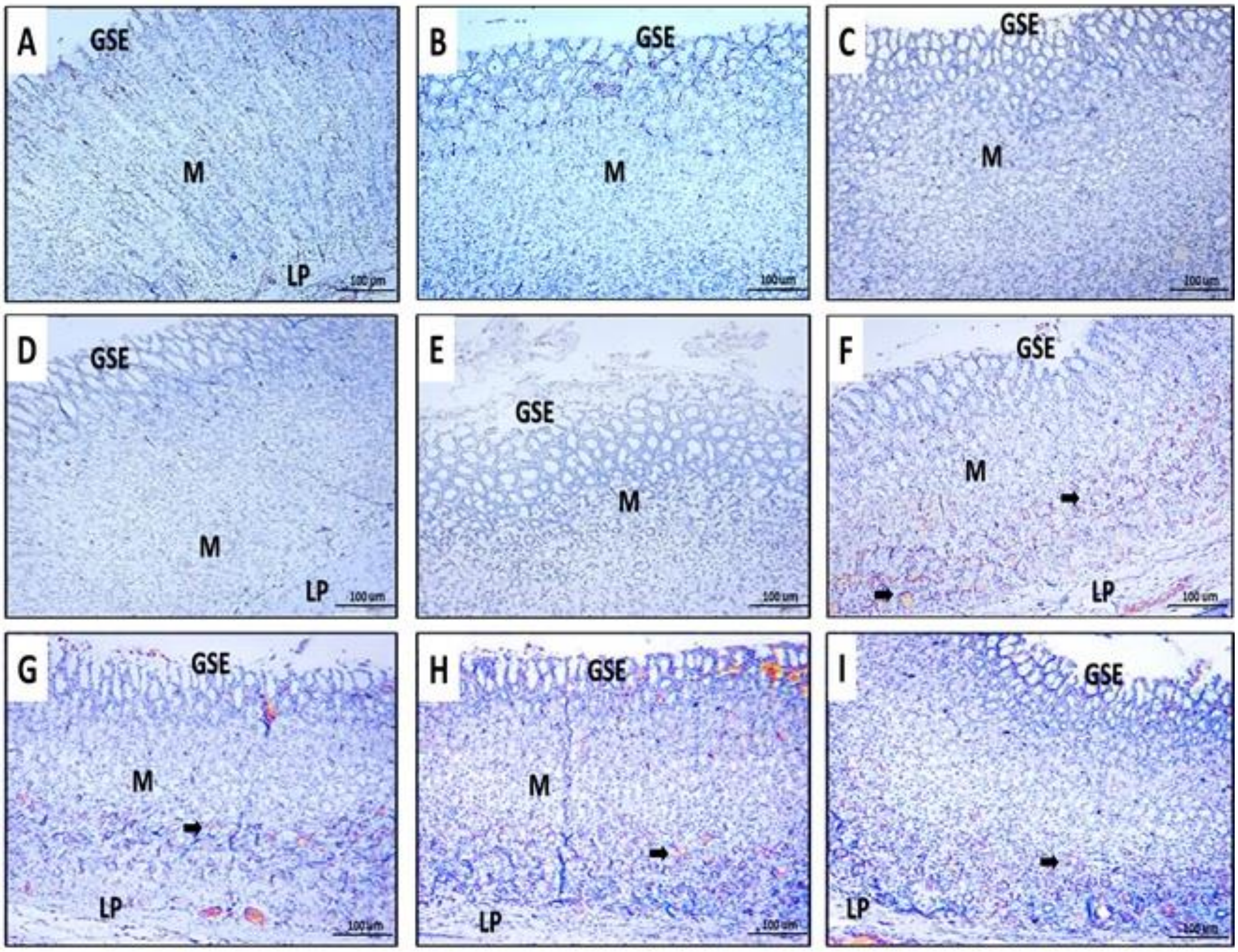
A
GSE
M
LP
B
GSE
M
C
GSE
M
D
GSE
M
LP
E
GSE
M
F
GSE
M
LP
G
GSE
M
LP
H
GSE
M
LP
I
GSE
LP

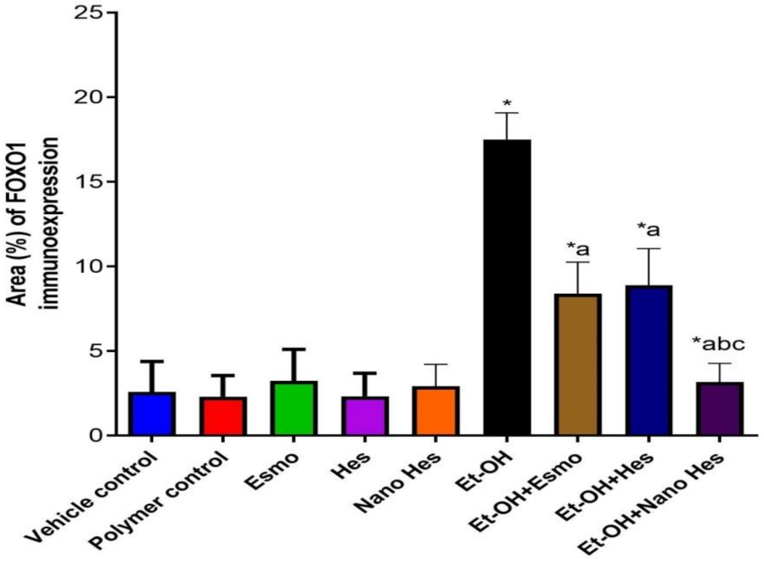
Area (%) of FOXO1 immunoexpression
0
5
10
15
20
25
*
*a
*a
*abc
Vehicle control
Polymer control
Esmo
Hes
Nano Hes
Et-OH
Et-OH+Esmo
Et-OH+Hes
Et-OH+Nano Hes

Figure S8. FOXO1 immunohistochemical analysis of rats' stomachs (200x, Scale bar = 100μm). FOXO1, a transcription factor involved in the regulation of oxidative stress and apoptosis, was assayed in the same experimental groups. In the control groups (Vehicle and Polymer), FOXO1 expression was found to be at baseline levels (Figures A-B), indicative of a stable cellular environment. Following treatments with Esmo, Hes, and Nano Hesperidin, there was no significant change in FOXO1 expression (Figures C-E), which could reflect a protective response against potential oxidative stress. Upon ethanol challenge, a significant increase in FOXO1 expression was detected (Figure F), suggesting a response to increased oxidative stress and potential apoptotic signaling. Notably, Esmo and Hesperidin pre-treatments resulted in a reduction of FOXO1 levels (Figures G-H), indicating mitigation of stress and apoptosis. The most substantial reduction was seen with Nanoemulsion Hesperidin pre-treatment, where FOXO1 levels were nearly normalized to those of the control groups (Figure I), highlighting its potent role in regulating stress responses and maintaining cellular homeostasis. Data expressed as mean ± SD (*n*= 6/group). [*] Compared to a vehicle control group, [a] Compared to ethanol-induced ulcerated rats group, [b] Compared to Esmo ulcerated group, and [c] Compared to free Hes ulcerated groups. Each group differed significantly from the others at $p \leq 0.05$. (M) mucosa, (G) glands, (I) isthmus, (N) neck, (B) base, (SGE) surface gastric epithelium, (SU) submucosa, (LP) lamina propria, (S) sloughing, (H) hemorrhage, (PC) parietal cells, (CC) chief cells, sloughed necrosis (SN), (bv) blood vessels.

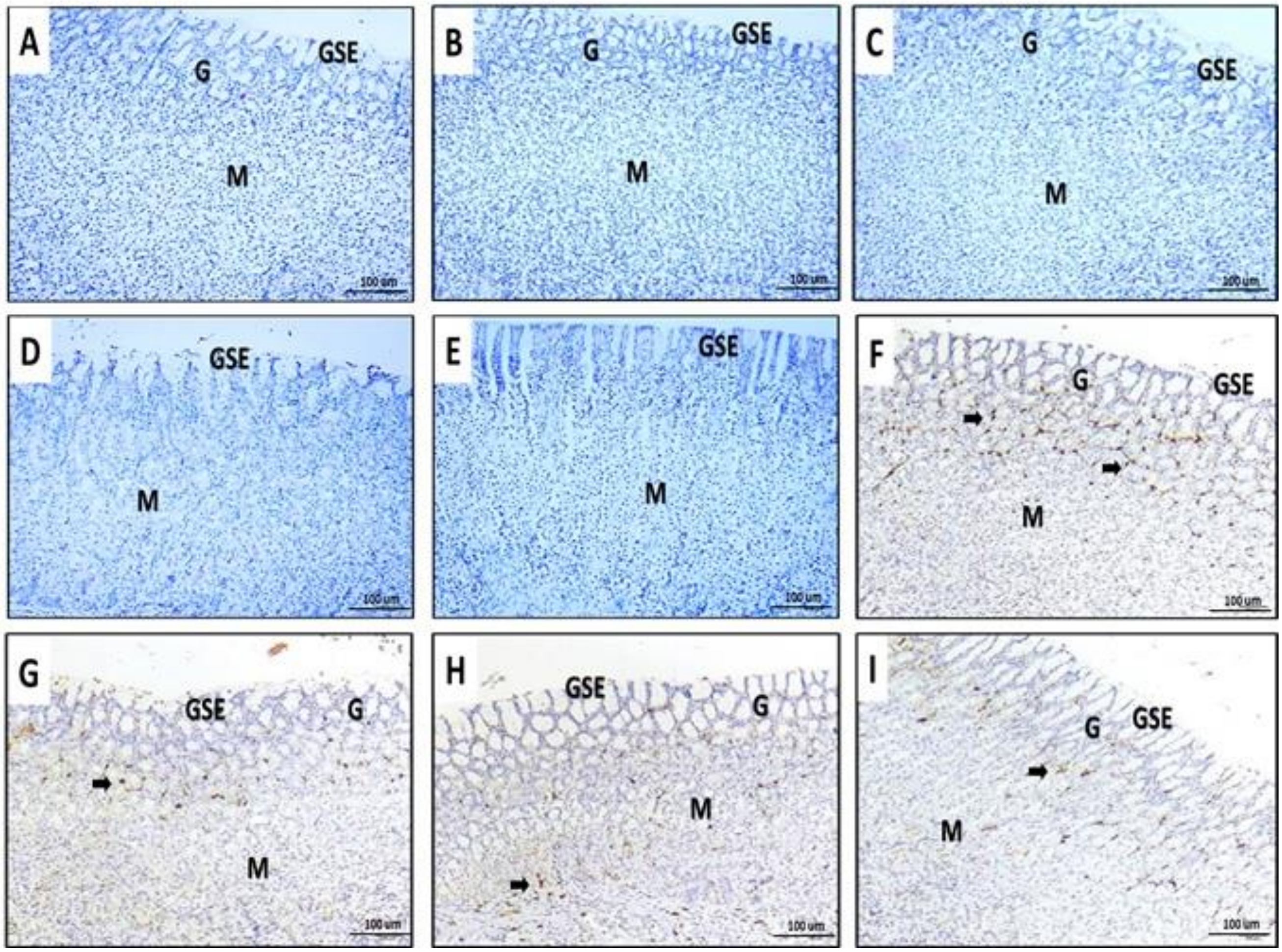
A
G
GSE
M
100 um
B
G
GSE
M
100 um
C
G
GSE
M
100 um
D
GSE
M
100 um
E
GSE
M
100 um
F
G
GSE
M
100 um
G
GSE
G
M
100 um
H
GSE
G
M
100 um
I
G
GSE
M
100 um

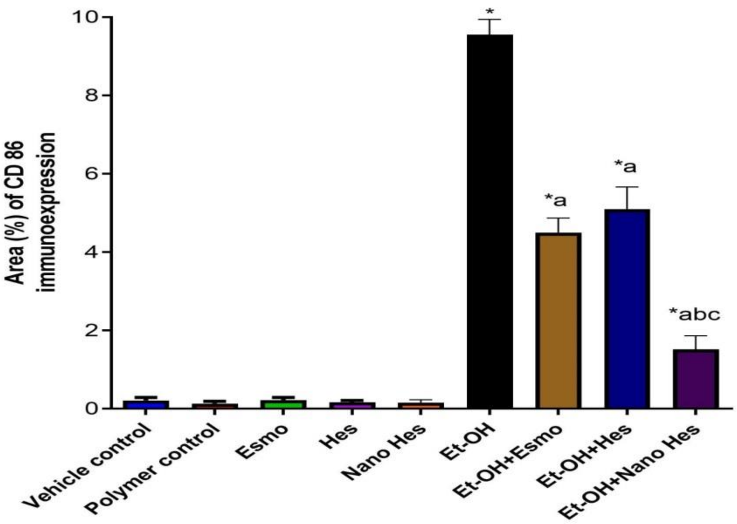
Area (%) of CD 86 immunoexpression
10
8
6
4
2
0
*
*a
*a
*abc
Vehicle control
Polymer control
Esmo
Hes
Nano Hes
Et-OH
Et-OH+Esmo
Et-OH+Hes
Et-OH+Nano Hes

Figure S9. CD86 immunohistochemical analysis of rats' stomachs (200x, Scale bar = 100µm). CD86, a marker for macrophage activation, was evaluated across different treatment groups. Under control conditions (Vehicle and Polymer), CD86 immunoreactivity was minimal, indicating low levels of macrophage presence or activation in the gastric tissue (Figures A-B). In the groups treated with Esmo, Hes, and Nanoemulsion Hesperidin, CD86 expression remained minimal, akin to the control conditions observed in the Vehicle and Polymer groups (Figures C-E). This suggests that these therapeutic interventions did not significantly alter the baseline levels of macrophage presence or activation within the gastric tissue. Remarkably, the ethanol-induced ulceration led to a pronounced upsurge in CD86 expression (Figure F), reflecting an intense macrophage-mediated inflammatory response. Pre-treatment with Esmo and Hesperidin somewhat moderated this increase (Figures G-H), whereas the Nanoemulsion Hesperidin pre-treatment significantly reduced CD86 expression, aligning closely with control levels (Figure I). This demonstrates the Nanoemulsion's efficacy in suppressing macrophage-driven inflammation. Data expressed as mean ± SD (*n*= 6/group). [*] Compared to a vehicle control group, [a] Compared to ethanol-induced ulcerated rats group, [b] Compared to Esmo ulcerated group, and [c] Compared to free Hes ulcerated groups. Each group differed significantly from the others at $p \leq 0.05$.
